# Editing metacaspase (*StMC7*) gene enhances late blight resistance in Russet Burbank potato

**DOI:** 10.1371/journal.pone.0325702

**Published:** 2025-06-18

**Authors:** Bikram Poudel, Atul Sathe, Jacqueline C. Bede, Ajjamada C. Kushalappa

**Affiliations:** 1 Plant Science Department, McGill University, Ste.-Anne-de-Bellevue, Quebec, Canada; ICAR-National Rice Research Institute, INDIA

## Abstract

Plants induce hypersensitive response programmed cell death (HR-PCD), upon biotrophic pathogen infection, to contain the pathogen to the point of infection. Apoptotic-like PCD (AL-PCD) has been reported upon prolonged hemibiotrophic and necrotrophic pathogen infection in potato, to feed on the dead cells for their growth. In potato, silencing of the gene *StHRC* lead to the suppression of AL-PCD, thus increasing resistance to blights in potato. This was also associated with a significant reduction in the expression of the metacaspase gene *StMC7*. Accordingly, the gene *StMC7* was silenced in potato cultivar ‘Russet Burbank’ using CRISPR-Cas9 to improve disease resistance against late blight of potato caused by *Phytophthora infestans*. Following pathogen infection, the disease severity, pathogen biomass and *StMC7* gene expression was lower in *Stmc7* mutants as compared to wild type. Disease severity was also decreased in *Alternaria solani* inoculated *Stmc7* mutants, compared to the wild type, suggesting possible multiple disease resistance in the *Stmc7* knockdown mutants. This confirms that the silencing of *StMC7* improves late blight disease resistance in potato.

## Introduction

Potato is one of the most widely consumed food crops grown around the world under different agroecological environments. Potato cultivation is continually threatened by pathogens that destroy the quality and quantity of potato yield. Major pathogens in potato are the oomycete pathogen *Phytophthora infestans* causing late blight. The management strategy to counter pathogens involves application of chemical pesticides, but this increases the cost of production and is also associated with human health and environmental hazards [1]. Improving host genetic resistance through development of disease resistance cultivars is the most sustainable and effective management strategy. Genome editing can improve disease resistance in commercial or elite breeding potato cultivars with less effort, time, as well as enhanced precision and efficiency [2]. It is important to understand the molecular mechanisms behind the resistance and susceptibility to pathogen attack for genome editing in potato.

Upon pathogen detection, plants immediately activate programmed cell death (PCD), initially in the form of hypersensitive response (HR), which is termed HR-PCD [3]. HR is a localized plant cell death at the infection point to restrict the food supply to the pathogen, thus limiting its spread to the point of infection. True apoptosis is absent in plants, unlike in animals, but apoptotic-like PCD (AL-PCD), which is morphologically and biochemically similar to apoptosis in animals, has been reported [4]. AL-PCD is induced later than HR-PCD, but it is still a rapid process, initiating and often ending within 6 h [5]. The DNA ladders, a characteristic feature of AL-PCD, appear in 6 hpi of pathogen detection [6]. Though both HR-PCD and AL-PCD are characterized by cytoplasmic shrinkage, mitochondrial swelling, cytochrome c release, chloroplast disruption, chromatin condensation and DNA fragmentation, they differ in the presence of plasma membrane blebbing and a characteristic DNA fragmentation [7]. Both HR and AL-PCD are detrimental to biotrophic pathogens as PCD limits its access to living cells and cuts off the nutrient source. However, hemibiotrophic and necrotrophic pathogens actively promote PCD in their host cells and utilize it to derive nutrients from the dead cells. Delaying or preventing AL-PCD can stop cell death, limiting the nutrient source for pathogens, resulting in enhanced plant resistance.

For pathogen detection, the pathogen-associated calcium ion (Ca^2+^) influx signals are perceived by calcium sensors such as calmodulin (CaM), CaM-like proteins, Ca^2+^ -dependent protein kinases (CDPKs) and Ca^2+^- and Ca^2+^/CaM-independent protein kinase (CCaMK) and calcineurin B-like proteins (CBLs) [8]. The pathogen-associated Ca^2+^ influx is perceived by the sarcoplasmic/endoplasmic histidine-rich Ca^2+^ -binding protein (HRC), increases the Ca^2+^ concentration in the cytosol, and transports to the nucleus further increases the concentration in the nucleus [9]. HRC, initially identified in fusarium head blight (FHB) resistant wheat near isogenic lines (NIL), seems to play an important role in Ca^2+^ mediated defense response [10]. Mutation in HRC, both naturally and through gene editing, leads to disease resistance; FHB resistance in wheat and blight resistance in potato. In wheat, *TaHRC* was found to be mutated in FHB resistant lines, while in potato, *StHRC* was silenced, where both lead to high resistance against blight diseases through suppression of AL-PCD [11–13].

Metacaspases mediate PCD in plants during development, and during abiotic and biotic stresses resistance and play important roles during AL-PCD [14,15]. Two types of metacaspases are found in plants, type I metacaspases, which contains an N-terminal prodomain and a subunit p20, and type II metacaspases, which contains a linker joining two subunits, p20 and p10 [16]. Changes in Ca^2+^ concentration affect the activity of metacaspases and endonucleases. Upon increased Ca^2+^ influx, metacaspases undergo conformational changes and is activated by multiple cleavage in the linker region of type II metacaspases [17]. *AtMC2d*, the most abundant type II metacaspases in Arabidopsis, exhibits Ca^2+^ dependency for activation [18]. Metacaspases then processes substates such as Propep1, producing Pep1 elicitors, which trigger downstream immune response and signal nearby cells [19]. Among the 9 metacaspases in potato, *StMC7*, showing significant similarity to *AtMC4* in arabidopsis, is constitutively highly expressed in most plant tissues [20]. Silencing of *StHRC* also reduced the expression of *StMC7* and increased the disease resistance to *Phytophthora infestans* and *Alternaria solani* [13]. Accordingly, it was hypothesized that the silencing of *StMC7* may also lead to the suppression of AL-PCD, thus inhibiting the infection process by hemibiotrophic and necrotrophic pathogens, due to suppression of their nutritional source, leading to increased plant resistance.

Here, we report *Solanum tuberosum* metacaspase 7 (*StMC7*) as a hemibiotrophic pathogen susceptibility gene, based on plant-pathogen interactions with the hemibiotrophic pathogen *Phytophthora infestans*. CRISPR/Cas9 mediated silencing of *StMC7* in Russet Burbank potato showed reduction in gene expression, disease severity and pathogen biomass. Disease severity for the necrotrophic pathogen *Alternaria solani* was also reduced in *Stmc7* mutants. Based on these findings, we report that silencing of the functional *StMC7* leads to enhanced disease resistance to late blight and may also provide multiple disease resistance in potato.

## Materials and methods

### Plant production

Tissue cultured plantlets of the susceptible potato genotype Russet Burbank (RB) was obtained from the New Brunswick Plant Propagation Center (Potato Research Centre, Agriculture and Agri-Food Canada, New Brunswick, Canada). The tissue culture plantlets were further multiplied in the lab and then grown in the greenhouse in pots with Fafard AGRO MIX® G6 under greenhouse condition (20 ± 3^o^C temperature, 70 ± 10% relative humidity, 16 h photoperiod, 1500 µmol/m^2^/s light intensity). The tissue cultured plantlets were grown in sterile half strength M516 media, supplemented with 3% sucrose and 2g/L Phytagel™. Plantlets were incubated in a Percival growth chamber at 22^o^C with 16 h light cycle. Internodal segments and leaf segments from 3-week-old sterile plants were used for *Agrobacterium*-mediated transformation.

### DNA extraction and *StMC7* sequencing

DNA was extracted from RB potato seedlings using a modified CTAB method [21]. The full gene sequence of *StMC7* covering both exons was amplified with specific primers using a high fidelity Q5 Polymerase (New England Biolabs, Ipswich, MA, USA) (Table 1). The amplified PCR product was purified using BioBasic PCR Clean Up (Bio Basic INC., Markham, ON, CA). The purified PCR product was cloned into pGEMT-Easy plasmid (Promega Corp., Madison, WI, USA) using T4 DNA Ligase (NEB, MA, USA). The final plasmid was transformed into competent *E. coli* DH5α cells (New England Biolabs, Ipswich, MA, USA) and sequenced using M13-F and R primers by Sanger sequencing (Institute of Integrative Biology and Systems (IBIS), Laval University) sequences were translated with Expasy (https://www.expasy.org/) to obtain protein sequence [22]. The protein structure of *StMC7* was predicted using RoseTTAFold [23].

**Table 1:** Primers used for gRNA synthesis, amplicon sequencing, pathogen biomass quantification, qRT-PCR.

Gene name	Primer sequence
sgRNA-1	F: 5’- GATTGAATATACGTAAAGCTTTAT-3’ R: 5’-AAACATAAAGCTTTACGTATATTC-3’
sgRNA-2	F: 5’- GATTGAGCTATGCGAAACCAGCCA-3’ R: 5’-AAACTGGCTGGTTTCGCATAGCTC-3
sgRNA-3	F: 5’- GATTGCAGATGCCACCCCTGCAGG-3’ R: 5’-AAACCCTGCAGGGGTGGCATCTGC-3’
*StMC7*-gRNA-1	F: 5’- GCGAAAAAGGCAGTGTTAATTGG-3’ R: 5’- CCCCCAACAAATTCAACAAACAC-3’
*StMC7*-gRNA-2–3	F: 5’- TGGGAAACTTAGGCCAACAC-3’ R: 5’- AGGCATAGCACAGCAGAATC-3’
AtCas9	F: 5’- CGCATCTCTCGGAACCTACC-3’ R: 5’- CCTCCCCTCTCAGCCTTAGT-3’
NGS-tag	F:5’-ACACTCTTTCCCTACACGACGCTCTTCCGATCT-3’ R:5’-GTGACTGGAGTTCAGACGTGTGCTCTTCCGATCT-3’
*StEf1-α*	F: 5’- ATTGGAAACGGATATGCTCCA −3’ R: 5’- TCCTTACCTGAACGCCTGTCA −3’
*Stβ-tubulin*	F: 5’- ATGTTCAGGCGCAAGGCTT-3’ R: 5’- TCTGCAACCGGGTCATTCAT-3’
*O-8(Phytophthora infestans*)	F: 5’- TGGGAAACTTAGGCCAACAC-3’ R: 5’- TAACCGACCAAGTAGT AAA −3’
*StMC7*-RT	F: 5’- GCCTTCTCGAGTAGCTGTTGA −3’ R: 5’- TCACATGATGGAGAATGGTT-3’

### Vector construction and *Agrobacterium*-mediated transformation

Three guide RNAs (gRNAs) were designed based on the *StMC7* gene sequence, targeting both exons, one targeting the first exon and two targeting the second exon, using CRISPR-P 2.0 (http://crispr.hzau.edu.cn/CRISPR2/) [24]. The three gRNAs were individually cloned into three separate pDIRECT21A vectors through Golden Gate Assembly using *AarI* restriction enzyme [25]. The final vectors were transformed into *E. coli* DH5α competent cells and sequenced (IBIS, Laval University). The clones with successfully inserted gRNA were transformed using the standard freeze-thaw method into *Agrobacterium tumefaciens* (GV3101) for explant transformation.

Transformation was carried out according to Duan et al. (2012) with slight modifications [26]. Briefly, internodes and leaves were sterilized using 70% ethanol for 30s followed by 50% NaOCl solution for 10 min for internodes and 5 min for leaves. The explants were subjected to a mix of three *Agrobacterium* suspension cultures harboring the three different constructs in pDIRECT_21A construct. The explants were transferred to co-cultivation media (1/10 MS salts, 3% sucrose, pH 5.7, 6g/L agar) at 22^o^C for 2 days and then transferred to callus induction media (CIM) (4.3 g/L MS salts, 2.5 mg/L zeatin, 0.1 mg/L naphthalene acetic acid (NAA), 3% sucrose. 6 g/L agar) with timentin (150 mg/L) and hygromycin (50 mg/L) and incubated at 22^o^C. After 1-month, growing calli were transferred to shoot induction media (SIM) (4.3 g/L MS media, 2.5 mg/L zeatin, 0.3 mg/L gibberellic acid, 6 g/L sugar, 3% sucrose) with timentin (150 mg/L) and hygromycin (50 mg/L). Calli with emerging shoots were transferred to MS media supplemented with 3% sucrose, 6 g/L agar, supplemented with previous dose of timentin and hygromycin.

### Detection of putative CRISPR-Cas9 potato mutants

Initial screening of transgenic seedlings was conducted through a PCR with Cas9 primers (Cas9-F and Cas9-R) (Table 1) using Phusion Green Hot Start II High-Fidelity PCD Master Mix (ThermoFisher Scientific, MA, USA) and run on 1% agarose gel, revealing a positive 957-bp band for Cas9 positive seedlings. A 526-bp surrounding the *StMC7* gRNA1 region was amplified with primer *StMC7*-gRNA-1 using Phusion Green Hot Start II High-Fidelity PCD Master Mix (ThermoFisher Scientific, MA, USA) and purified using the BioBasic PCR Purification Kit (Biobasic, ON, CA). The purified sample was subjected to restriction digestion assay using *Hind*III, which cut at a unique site within the gRNA 1, as per the manufacturer’s protocol. A barcoding library was prepared using tags NGS-tag-F + *StMC7*-gRNA-1-F and NGS-tag-R + *StMC7*-gRNA-1-R. The library was purified and sent for sequencing by Illumina NextSeq PE300-500K at Genome Quebec, Montreal. Results from amplicon sequencing were analyzed using CRISPResso2 [27].

### Disease severity and pathogen biomass assay

*Phytophthora infestans* isolate US-8, A2 mating type, a highly virulent, aggressive strain, (received from Dr. H. Platt, AAFC, Charlestown, PEI, CA) was maintained on potato dextrose agar (PDA). Spores were produced by inoculating a thin potato tuber slice with *P. infestans* and incubating the sealed and moist Petri dish in 18^o^C for sporulation. Sporangia were harvested and spore concentration was adjusted to 10^5^ sporangia per ml. *Alternaria solani* (obtained from Dr. A. Dionne, MAPAQ, QC) was maintained on PDA. For spore production, the plate was incubated at room temperature (21 – 23^o^C) with 12 h light and dark photoperiod.

The experiment for pathogen biomass and disease severity was conducted in a Randomized Complete Block Design (RCBD) with two genotypes (wild RB and *StMC7* mutants), two inoculations (mock and pathogen), and three temporal replicates. Each experimental unit consisted of five pots with two plants and ten leaves inoculated in each pot. Young leaves of 5–6 weeks old plants were point inoculated on the either side of midrib on the lower surface with 10 µl sporangial suspension. The plants were covered with plastic bags upon inoculation for 72 h and 48 h for *P. infestans* and *A. solani*, respectively, and kept in greenhouse at 21-23^o^C. Disease severity was quantified by measuring the lesion diameter (in mm) at 3 days interval until 9 post-inoculation (dpi) and calculating the area under the disease progress curve (AUDPC). Relative biomass for *P. infestans* from infected samples was quantified based on quantitative PCR (qPCR) to determine the biomass of the pathogens in infected samples. Genomic DNA was isolated from the infected leaves 6 dpi using the CTAB method. Specific primers for *P. infestans*, as well as against the potato genome was used in qPCR performed using a Luna Universal qPCR Mastermix (NEB, MA, USA) using Mic qPCR Cycler (Bio Molecular Systems, Queensland, Australia) (Table 1) [28]. Statistical analysis was conducted using SPSS (IBM SPSS Statistics 29.0).

### RNA extraction and gene expression by RT-qPCR

NCBI BLAST was used to design RT-qPCR primers [29]. RNA was extracted from the leaves of RB genotype and *Stmc7* knockdown plants 6 days post inoculation (dpi) following *P. infestans* and mock (water) inoculation using RNeasy Plant Mini Kit (Qiagen, Venlo, Netherlands). cDNA was synthesized using Maxima H Minus First Strand cDNA Synthesis Kit (ThermoFisher Scientific, MA, USA). qPCR was conducted with Luna Universal qPCR Mastermix (NEB, MA, USA). Two genes, elongation factor alpha (*StEf1α)* and *tubulin* (*Sttubulin*) were used as reference genes. The relative gene expression levels were analyzed based on delta-delta C_T_ (cycle threshold) method [30].

## Results

### Characterization of *StMC7*

**Fig 1:**
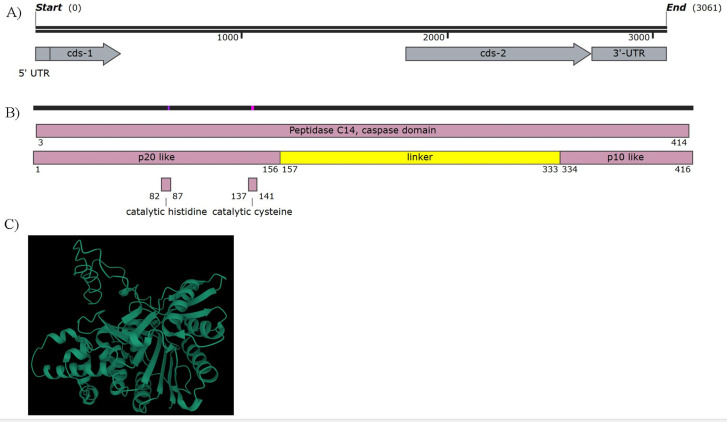
Characterization of the potato metacaspase StMC7. (A) gene structure showing the two coding sequence regions, corresponding to the p20 and p10 domain associated with type II metacaspases. **(B)** StMC7 protein structure with the domains, p20 and p10 region, linker region and the conserved catalytic histidine and cysteine within the p20 region. **(C)** Predicted protein structure of StMC7 obtained using RoseTTAFold.

The *StMC7* gene was found to be 3061 bp, with two exons, 346 bp and 905 bp (Fig 1A). The gene sequence has been submitted to NCBI GenBank (Accession number PQ811591). Protein sequence analysis revealed the presence of a peptidase C14 caspase domain, supporting the role of *StMC7* as a metacaspase (Fig 1B). Upon comparison with arabidopsis *AtMC4* sequence, the exact positions for conserved caspase-like catalytic domains such as the p-20 subunit (20 kDa), p-10 subunit (10 kDa) and linker between them were identified. The catalytic histidine was found within the sequence HYSGHG and the catalytic cysteine was found within DSCHS, similar to *AtMC4* (Fig 1B) [31]. The protein structure of *StMC7* was predicted using RoseTTAFold, revealing its catalytically inactive state (Fig 1C).

### *StMC7* silencing based on CRISPR-Cas9

**Fig 2:**
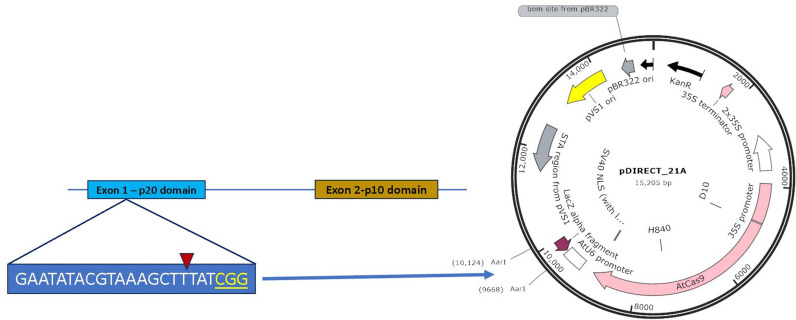
CRISPR design and construct preparation. gRNA was selected from the Exon 1 of StMC7 and introduced into the pDIRECT_21A vector using Golden Gate Assembly at the AarI position of the vector to prepare CRISPR construct.

**Fig 3:**
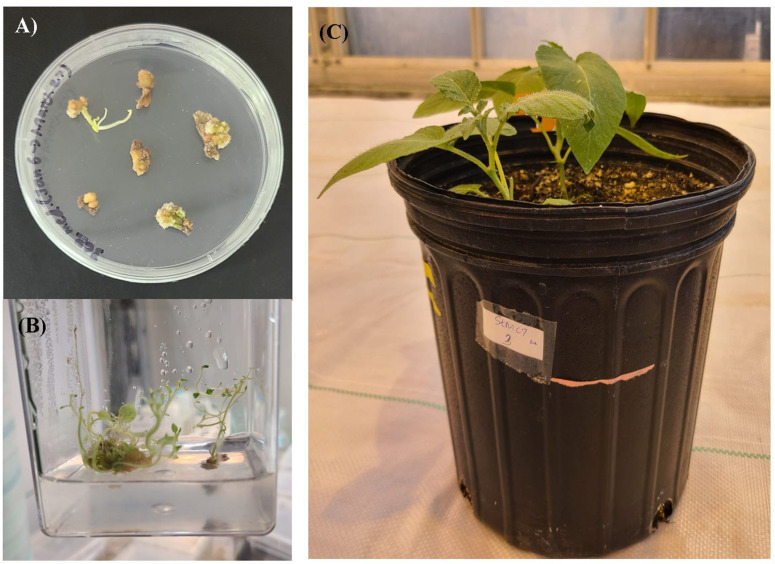
Stmc7 mutant regeneration from callus regeneration of internodes and leaves of RB potato using Agrobacterium-mediated transformation for CRISPR construct delivery. **(A)** Regenerating plantlets after Agrobacterium-mediated transformation of infected leaves and internodal segments. **(B)** Transgenic seedlings growing in shoot regeneration media **(C)** Stmc7 mutant.

**Fig 4:**
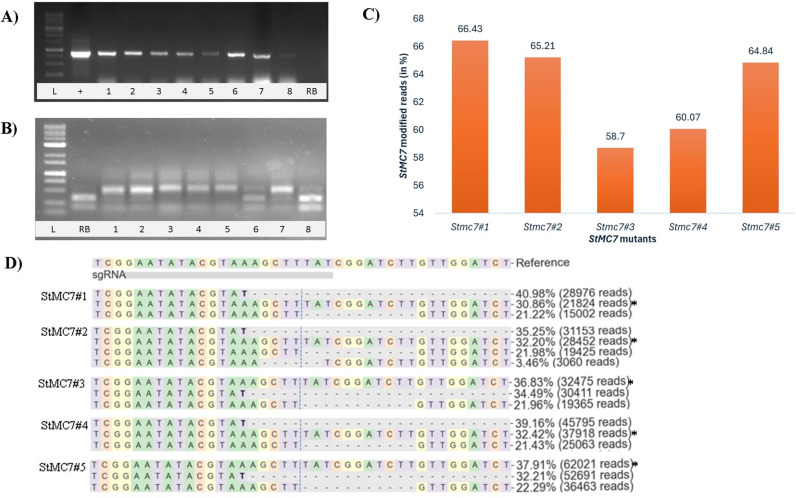
CRISPR-Cas9-mediated mutation analysis of T_0_ Stmc7 mutants. **(A)** Confirmation of Cas9 presence in all eight regenerated potato seedlings through a 957 bp band. L: 1 kb plus ladder, + : Positive Control (pDIRECT21A plasmid), 1-8: Stmc7 transgenic samples, RB: wild type. **(B)** HindIII digestion assay to identify Stmc7 mutants. L: 1 kb plus ladder, RB: Positive Control, 1-8: Stmc7 transgenic samples. Wild type RB (lane RB) or non-edited samples (lane 6,8) gives two bands. **(C)** Percentage of total modified reads for each Stmc7 mutant line. The value at the top of the bar represents the exact percentage of modified reads. **(D)** Indels detected in Stmc7 mutants by targeted amplicon sequencing mapped to wild type sequence. The percentage of reads for each sequence and no. of reads with that sequence are shown on the right. Sequences below 2% are not shown. The asterisk (*) indicates non-edited reads. Blue vertical dash line indicates predicted cleavage position, black horizontal dash indicates base deletion.

Plants with InDel mutations were generated targeting the *StMC7* exon 1 through *Agrobacterium*-mediated transformation of the CRISPR-Cas9 construct (Fig 2) in the late blight susceptible cultivar Russet Burbank (RB). Transformed internodes and leaves successfully produced callus under the selection of hygromycin, resulting in putative transgenic potato plants (Fig 3). Eight plants were obtained after hygromycin selection through every stage of tissue culture. PCR and gel analysis of all regenerated eight seedlings exhibited the presence of Cas9 (Fig 4A). Restriction digestion assay showed InDel mutations in 6 plants at gRNA 1 (Fig 4B). No mutations were observed upon Sanger sequencing for gRNA 2 and 3. Amplicon-sequencing followed by CRISPResso2 analysis determined five heterozygous mutants in exon 1 of *StMC7* (Fig 4C).

### *Stmc7* reduced disease severity and pathogen biomass in Russet Burbank potato

**Fig 5:**
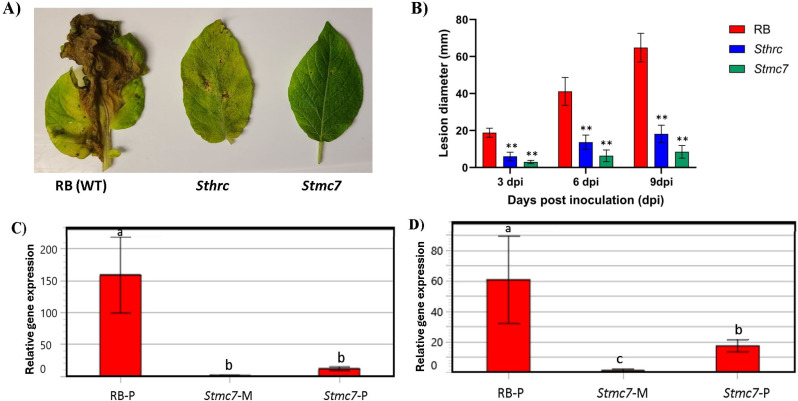
Russet Burbank (RB) potato leaves, with functional and mutated metacaspase Stmc7, inoculated with Phytophthora infestans. **(A)** Disease progression based on the lesion diameter (mm) at 3-, 6- and 9-days post inoculation (dpi) **(B)** Late blight symptoms showing highly significant differences between RB and Stmc7 mutants (p < 0.001), significant differences between StHRC and StMC7 mutants (p = 0.02). **(C)** Pathogen biomass quantification at 6 days post-inoculation, quantified as relative P. infestans specific (O-8) gene expression **(D)** Relative gene expression of StMC7 in RB and Stmc7 mutants compared to the reference genes StEf1 and tubulin, relative to wildtype mock inoculation, following Phytophthora infestans inoculation. Significance determined by Student’s t-test, P value: **p < 0.01, *p < 0.05, or multiple comparison test (Tukey’s HSD), significant differences shown by lowercase letters (p < 0.05). Error bars represent mean standard error (SEM) and asterisks denote values significantly different to wild type (p < 0.05).

**Fig 6:**
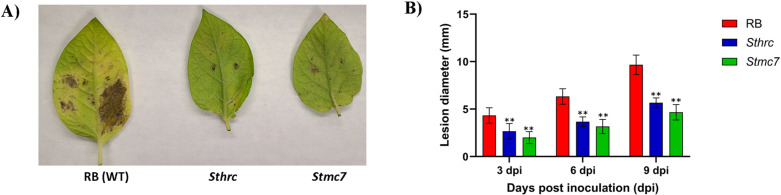
Russet Burbank potato leaves, with functional and mutated metacaspase Stmc7, infected with Alternaria solani. **(a)** Disease progress based on the lesion diameter (mm) at 3-, 6- and 9-days post inoculation **(b)** Early blight symptoms showing differences between RB and Stmc7 mutants.

Disease severity was assessed among *Stmc7*, and *Sthrc* mutants, and RB non-edited as control. Though circular lesions began to appear in all samples from 3 dpi for both early and late blight diseases, the lesions in RB control were much larger than *Stmc7* and *Sthrc*. The area under disease progress curve (AUDPC) was seven-fold lower in *Stmc7* mutants (36.25) as compared to RB (249) and two-fold lower than *Sthrc* mutants (77.25). *StMC7* was upregulated in pathogen-infected RB wildtype plants compared to the *Stmc7* mutants for *P. infestans* inoculated leaves (Fig 5D). Pathogen biomass in the infected leaves with *P. infestans* specific primers quantified based on qPCR at 6 dpi were significantly higher in RB compared to the *Stmc7* mutants (fold change = FC = 13.53) (Fig 5), which reflects lower *P. infestans* levels in *Stmc7* mutants compared to the RB wildtype (Fig 5C). *StMC7* gene expression was lower in *Stmc7* mutants for *P. infestans* (FC = 3.47) inoculated leaves compared to pathogen inoculated RB plants (Fig 5D). In *A. solani* infected leaves, the AUDPC, calculated based on lesion diameter, was twice as low in *Stmc7* mutants (19.5) compared to RB (43.5) (Fig 6).

## Discussion

Programmed cell death (PCD) is an important defense strategy against biotrophic pathogens in plants. Hemibiotrophic oomycetes, such as *Phytophthora infestans* have a short, sometimes cryptic, biotrophic phase before switching to necrotrophic phase [32]. These pathogens induce effectors to elicit effector triggered immunity (ETI) and several elicitors or molecular patterns to elicit pattern triggered immunity (PTI) in plants, leading to HR-PCD. *Phytophthora infestans* produces fatty acids, such as Arachidonic acid, apoplastic effectors, such as necrotizing toxins Nep1-like proteins (NLPs), PcF-like (*Phytophthora cactorum* in *Fragaria*-like) small cysteine-rich proteins (SCRs)), and cytoplasmic effectors, such as CRN (crinkling, necrosis), etc. to induce AL-PCD [33,34]. Necrotrophic pathogen such as *Alternaria* spp. produces non-host specific toxins such as alternariol, zinniol, tentoxin and host-specific toxin families to elicit host defense response suppression and stimulate cell death [35]. This induction of cell death through PCD in plants is important in increasing food supply for necrotrophic and hemibiotrophic pathogens [36]. Suppressing cell death by silencing host PCD pathway genes can confer resistance to such pathogens [13].

In this study, a key PCD pathway gene, metacaspase 7 (*StMC7*) was silenced using CRISPR gene editing technology, generating *Stmc7* mutants with InDel mutations in exon 1. The reduction in the AUDPC for both early and late blights possibly indicate successful suppression of pathogen attack in the mutants, which can be attributed to reduced PCD by host upon pathogen infection due to silencing of *StMC7*. For *P. infestans*, there were lower pathogen growth in *Stmc7* mutants compared to control RB plants (Fig 5C). For *A. solani*, disease progression was twice as slow in *Stmc7* mutants compared to the wild type. Further experiments under field conditions are necessary to confirm the *Stmc7* mutants’ response to *P. infestans* and *A. solani* infection.

Metacaspases such as *StMC7* have been involved in plant pathogen resistance in arabidopsis, tomato, wheat, and pepper [16]. In arabidopsis, mutations in type 2 metacaspases, *AtMC2* to *AtMC6* resulted in reduced susceptibility to *Botrytis cinera* and *B*. *tulipae* [37]. In tomato, *LeMC1* was rapidly induced when infected by *B. cinera* [38]. In wheat, the expression of *TaMC4* was significantly upregulated when challenged with *Puccinia striiformis* f.sp. *tritici*. Virus-induced gene silencing (VIGS) of *TaMC4* resulted in limited fungal growth [39]. Another metacaspase, *TaMC1* was significantly upregulated upon infection by *Puccinia striiformis* and increased disease resistance upon knockdown of *TaMC1* expression through VIGS [40]. VIGS of metacaspase 9 in pepper (*CaMC9*) lead to delayed cell death symptoms and reduced cell death induced by *Xanthomonas campestris* pv*. vesicatoria* while the overexpression of *CaMC9* enhanced cell death and increased susceptibility to *Pseudomonas syringae* pv*. tabaci* [41]. In potato, *StMC7* was significantly downregulated upon silencing histidine-rich calcium-binding protein coding gene (*StHRC*) indicating its prominent role in inducing AL-PCD [13].

### Model for AL-PCD induction upon pathogen attack

**Fig 7:**
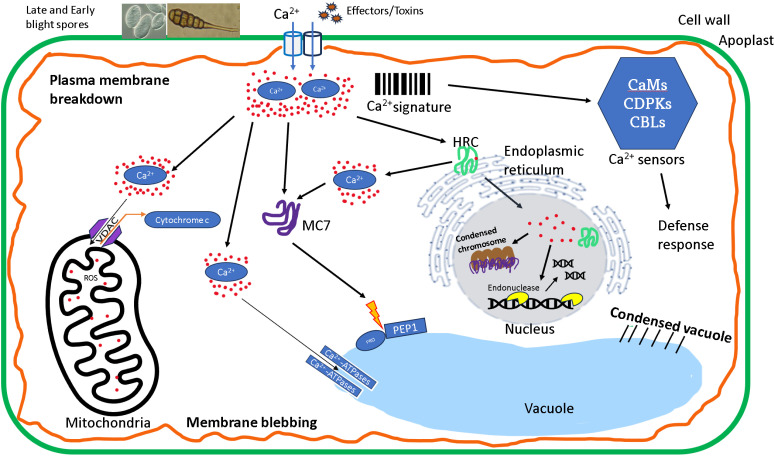
Proposed model of apoptotic-like programmed cell death (AL-PCD) in plants. Upon Ca^2+^ entry associated with pathogen attack, Ca^2+^ sensors decode the calcium signal for downstream activation. *StHRC* (histidine-rich calcium-binding protein) binds with Ca^2+^ [13] and moves into the nucleus, increasing the calcium concentration in nucleus which activates endonucleases and causes DNA fragmentation. Ca^2+^ also enters the mitochondria through VDAC (voltage-dependent anion channel), which produces ROS in mitochondria. Ca^2+^ activates metacaspases *StMC7* in cytosol. The *StMC7* then cleaves PROPEP1, releasing PEP1, which signals nearby cells to initiate damage response and leads to condensed vacuole and other organelles. The major characteristics of AL-PCD are shown in bold letters: Plasma membrane breakdown, condensed organelles such as vacuole, mitochondria, condensed chromosome, membrane blebbing.Abbreviations: CaMs: calmodulins, CDPKs: Calcium-dependent protein kinases, CBLs: calcineurin B-like proteins.

Though AL-PCD is induced during plant-pathogen interaction, the exact pathway of induction is not clear. An essential condition for metacaspases activation is an increase in Ca^2+^. A model on the pathway of AL-PCD induction in potato has been proposed [13], and a modified version involving *StMC7* combining the role of *StHRC* is shown in Fig 7. In general, an influx of intracellular Ca^2+^ following fungal pathogen is sensed by various Ca^2+^ influx protein sensors responsible for maintaining cytosolic Ca^2+^ homeostasis [42]. Ca^2+^ -transporters such as Ca^2+^ -ATPases, two-pore Ca^2+^ channels and cyclic nucleotide gated channels (CNGCs), transport Ca^2+^ out of cytosol, across various cellular membranes [43]. When Ca^2+^ ion concentration increases in mitochondria, cytochrome c is released via the formation of permeability transition (PT) pore or through the large cytochrome c-conducting channel formed by voltage-dependent anion channel (VDAC), increasing the reactive oxygen species (ROS) [44]. Ca^2+^ - binding proteins such as *StHRC* regulate the downstream response processes [45]. HRC transports the Ca^2+^ into nucleus, where the Ca^2+^ triggers Ca^2+^- dependent endonucleases such as *StCaN2*, to degrade DNA [13,46]. A higher concentration of Ca^2+^ in the cytosol triggers the activation of *StMC7* in the cytosol through multiple cleavage at 6 sites in the linker region releasing the linker region and making the active site available for substrate processing [17]. Structures such as apoptosome have been reported to activate caspases and execute AL-PCD in animals [47]. Cryo-electron microscopy may elucidate if similar structures may form before AL-PCD in potato. Silencing genes involved in apoptosome, and other candidate genes involved in AL-PCD, endonucleases such as *StCaN2*, could also reduce the DNA fragmentation, induction of AL-PCD and provide further multiple disease resistance.

The mechanism of resistance through HR-PCD is the death of cells that limit food supply stopping the growth of a biotrophic pathogen. However, in AL-PCD the mechanism of resistance is quite complex. The dead cells increase food supply, increasing the growth of hemibiotrophic and necrotrophic pathogens. Whereas the silencing of AL-PCD leads to reduced disease severity, and the reduction is considered not to be due to resistance genes. However, the induction of AL-PCD increases pathogen biomass, which produces more toxins. These toxins may also suppress the expression of genes responsible for resistance, as observed in wheat infected with *Fusarium graminearum* which produces deoxynivalenol that inhibits the eukaryotic protein translation including enzymes catalyzing several resistance related (RR) metabolites important for reducing pathogen advancement through host cell wall accumulation and antifungal properties [48]. Thus, the silencing of AL-PCD not only reduces the susceptibility but also increases resistance by enabling the expression of resistance genes.

## Conclusion

Potato cultivars with multiple disease resistance are the desired outcome for all potato breeding programs. PCD is an important pathway in plant-pathogen interactions, which when suppressed may increase resistance to multiple pathogens. Upon pathogen- or damage-associated molecular pattern recognition, the plant activates the HR-PCD pathway. Apoptotic-like PCD has also been observed in plants, which involves plasma membrane blebbing and characteristic DNA fragmentation. AL-PCD generates nutrient sources for hemibiotrophic and necrotrophic pathogens. By silencing one of the important genes that mediate AL-PCD, a metacaspase *StMC7*, we have shown increased resistance against important potato pathogen, *Phythophthora infestans*, causing late blight and possible resistance to *Alternaria solani*, causing early blight of potato. Further experiments with other hemibiotrophic and necrotrophic pathogens on *StMC7* mutants should prove this to be a common multiple disease resistance mechanism in plants.
