# Supplementary material for: Editing metacaspase (StMC7) gene enhances late blight resistance in Russet Burbank potato
Source: PLoS One. 2025 Jun 18;20(6):e0325702. doi: 10.1371/journal.pone.0325702 (PMC12176156; doi:10.1371/journal.pone.0325702)

PLOS ONE

Editing metacaspase (StMC7) gene enhances late blight resistance in Russet Burbank potato

--Manuscript Draft--

**Manuscript Number:**	PONE-D-25-02422
**Article Type:**	Research Article
**Full Title:**	Editing metacaspase (StMC7) gene enhances late blight resistance in Russet Burbank potato
**Short Title:**	Editing metacaspase (StMC7) gene enhances late blight resistance in Russet Burbank potato
**Corresponding Author:**	Ajjamada C. Kushalappa, Ph. D.<br>McGill University<br>Ste.-Anne-de-Bellevue, Quebec CANADA
**Keywords:**	Gene editing, Programed cell death, Metacaspase, Disease resistance, Potato
**Abstract:**	Plants induce hypersensitive response programmed cell death (HR-PCD), upon biotrophic pathogen infection, to contain the pathogen to the point of infection. Apoptotic-like PCD (AL-PCD) has been reported upon prolonged hemibiotrophic and necrotrophic pathogen infection in potato, to feed on the dead cells for their growth. In potato, silencing of the gene StHRC lead to the suppression of AL-PCD, thus increasing resistance to blights in potato. This was also associated with a significant reduction in the expression of the metacaspase gene StMC7, in the AL-PCD pathway. Accordingly, the gene StMC7 was silenced in potato cultivar 'Russet Burbank' using CRISPR-Cas9 to improve disease resistance against late blight of potato caused by Phytophthora infestans. Following pathogen infection, the disease severity, pathogen biomass and StMC7 gene expression was lower in Stmc7 mutants as compared to wild type. Disease severity was also decreased in Alternaria solani inoculated Stmc7 mutants, compared to the wild type, suggesting possible multiple disease resistance in the Stmc7 knockout mutants. This confirms that the silencing of StMC7 improves late blight disease resistance in potato.
**Order of Authors:**	Bikram Poudel
	Atul Sathe
	Jacqueline Bede
	Ajjamada C. Kushalappa, Ph. D.
**Additional Information:**	
**Question**	**Response**
**Financial Disclosure**<br><br>Enter a financial disclosure statement that describes the sources of funding for the work included in this submission. Review the submission guidelines for detailed requirements. View published research articles from *PLOS ONE* for specific examples.<br><br>This statement is required for submission and **will appear in the published article** if the submission is accepted. Please make sure it is accurate.	Yes

Powered by Editorial Manager® and ProduXion Manager® from Aries Systems Corporation

**Funded studies**<br>Enter a statement with the following details:<br>• Initials of the authors who received each award<br>• Grant numbers awarded to each author<br>• The full name of each funder<br>• URL of each funder website<br>• Did the sponsors or funders play any role in the study design, data collection and analysis, decision to publish, or preparation of the manuscript?<br><br>Did you receive funding for this work?	
Please add funding details.<br>as follow-up to "**Financial Disclosure**<br><br>Enter a financial disclosure statement that describes the sources of funding for the work included in this submission. Review the submission guidelines for detailed requirements. View published research articles from *PLOS ONE* for specific examples.<br><br>This statement is required for submission and **will appear in the published article** if the submission is accepted. Please make sure it is accurate.<br><br>**Funded studies**<br>Enter a statement with the following details:<br>• Initials of the authors who received each award<br>• Grant numbers awarded to each author<br>• The full name of each funder<br>• URL of each funder website<br>• Did the sponsors or funders play any role in the study design, data collection and analysis, decision to publish, or preparation of the manuscript?<br><br>Did you receive funding for this work?"	Weston Loblaws and Natural sciences and engineering council of Canada
Please select the country of your main research funder (please select carefully as in some cases this is used in fee	CANADA - CA

calculation).<br>as follow-up to "**Financial Disclosure**<br><br>Enter a financial disclosure statement that describes the sources of funding for the work included in this submission. Review the submission guidelines for detailed requirements. View published research articles from *PLOS ONE* for specific examples.<br><br>This statement is required for submission and **will appear in the published article** if the submission is accepted. Please make sure it is accurate.<br><br>**Funded studies**<br>Enter a statement with the following details:<br>• Initials of the authors who received each award<br>• Grant numbers awarded to each author<br>• The full name of each funder<br>• URL of each funder website<br>• Did the sponsors or funders play any role in the study design, data collection and analysis, decision to publish, or preparation of the manuscript?<br><br>Did you receive funding for this work?"	
**Competing Interests**<br><br>Use the instructions below to enter a competing interest statement for this submission. On behalf of all authors, disclose any competing interests that could be perceived to bias this work—acknowledging all financial support and any other relevant financial or non-financial competing interests.<br><br>This statement is required for submission and **will appear in the published article** if the submission is accepted. Please make sure it is accurate and that any funding sources listed in your Funding Information later in the submission form are also declared in your Financial Disclosure	No authors have competing interests

statement.<br><br>View published research articles from *PLOS ONE* for specific examples.<br><br>**NO authors have competing interests**<br><br>Enter: *The authors have declared that no competing interests exist.*<br><br>**Authors with competing interests**<br><br>Enter competing interest details beginning with this statement:<br><br>*I have read the journal's policy and the authors of this manuscript have the following competing interests: [insert competing interests here]*<br><br>* typeset	
**Ethics Statement**<br><br>Enter an ethics statement for this submission. This statement is required if the study involved:<br><br>• Human participants<br>• Human specimens or tissue<br>• Vertebrate animals or cephalopods<br>• Vertebrate embryos or tissues<br>• Field research<br><br>Write "N/A" if the submission does not require an ethics statement.<br><br>General guidance is provided below. Consult the submission guidelines for detailed instructions. **Make sure that all information entered here is included in the Methods section of the manuscript.**	McGill University

**Format for specific study types**<br><br>**Human Subject Research (involving human participants and/or tissue)**<br>• Give the name of the institutional review board or ethics committee that approved the study<br>• Include the approval number and/or a statement indicating approval of this research<br>• Indicate the form of consent obtained (written/oral) or the reason that consent was not obtained (e.g. the data were analyzed anonymously)<br><br>**Animal Research (involving vertebrate animals, embryos or tissues)**<br>• Provide the name of the Institutional Animal Care and Use Committee (IACUC) or other relevant ethics board that reviewed the study protocol, and indicate whether they approved this research or granted a formal waiver of ethical approval<br>• Include an approval number if one was obtained<br>• If the study involved *non-human primates*, add *additional details* about animal welfare and steps taken to ameliorate suffering<br>• If anesthesia, euthanasia, or any kind of animal sacrifice is part of the study, include briefly which substances and/or methods were applied<br><br>**Field Research**<br><br>Include the following details if this study involves the collection of plant, animal, or other materials from a natural setting:<br>• Field permit number<br>• Name of the institution or relevant body that granted permission	
**Data Availability**<br><br>Authors are required to make all data underlying the findings described fully available, without restriction, and from the time of publication. PLOS allows rare exceptions to address legal and ethical concerns. See the PLOS Data Policy and FAQ for detailed information.	Yes - all data are fully available without restriction

A Data Availability Statement describing where the data can be found is required at submission. Your answers to this question constitute the Data Availability Statement and **will be published in the article**, if accepted.<br><br>**Important:** Stating 'data available on request from the author' is not sufficient. If your data are only available upon request, select 'No' for the first question and explain your exceptional situation in the text box.<br><br>Do the authors confirm that all data underlying the findings described in their manuscript are fully available without restriction?	
**Describe where the data may be found in full sentences. If you are copying our sample text, replace any instances of XXX with the appropriate details.**<br><br>• If the data are **held or will be held in a public repository**, include URLs, accession numbers or DOIs. If this information will only be available after acceptance, indicate this by ticking the box below. For example: *All XXX files are available from the XXX database (accession number(s) XXX, XXX.).*<br>• If the data are all contained **within the manuscript and/or Supporting Information files**, enter the following: *All relevant data are within the manuscript and its Supporting Information files.*<br>• If neither of these applies but you are able to provide **details of access elsewhere**, with or without limitations, please do so. For example:<br><br>*Data cannot be shared publicly because of [XXX]. Data are available from the XXX Institutional Data Access / Ethics Committee (contact via XXX) for researchers who meet the criteria for access to confidential data.*<br><br>*The data underlying the results presented in the study are available from (include the name of the third party*	All data included in the manuscript.

and contact information or URL).<br>• This text is appropriate if the data are owned by a third party and authors do not have permission to share the data.<br><br>* typeset	
Additional data availability information:	

Manuscript
Click here to access/download;Manuscript;pLOS-one-manuscript-2025.docx

Editing metacaspase (*StMC7*) gene enhances late blight resistance in Russet Burbank potato

Bikram Poudel[1¶], Atul Sathe[1¶], Jacqueline C Bede[1], Ajjamada C Kushalappa[1]*

[1] Plant Science Department. McGill University, Ste.-Anne-de-Bellevue, QC, Canada

* Corresponding Author: ajjamada.kushalappa@mcgill.ca

¶ These authors contributed equally to this work.

Abstract

Plants induce hypersensitive response programmed cell death (HR-PCD), upon biotrophic pathogen infection, to contain the pathogen to the point of infection. Apoptotic-like PCD (AL-PCD) has been reported upon prolonged hemibiotrophic and necrotrophic pathogen infection in potato, to feed on the dead cells for their growth. In potato, silencing of the gene *StHRC* lead to the suppression of AL-PCD, thus increasing resistance to blights in potato. This was also associated with a significant reduction in the expression of the metacaspase gene *StMC7*, in the AL-PCD pathway. Accordingly, the gene *StMC7* was silenced in potato cultivar 'Russet Burbank' using CRISPR-Cas9 to improve disease resistance against late blight of potato caused by *Phytophthora infestans*. Following pathogen infection, the disease severity, pathogen biomass and *StMC7* gene expression was lower in *Stmc7* mutants as compared to wild type. Disease severity was also decreased in *Alternaria solani* inoculated *Stmc7* mutants, compared to the wild type, suggesting possible multiple disease resistance in the *Stmc7* knockout mutants. This confirms that the silencing of *StMC7* improves late blight disease resistance in potato.

Introduction

Potato is one of the most widely consumed food crops grown around the world under different agroecological environments. The potato cultivation is continually threatened by pathogens that destroy the quality and quantity of potato yield. Major pathogens in potato are the oomycete pathogen *Phytophthora infestans* causing late blight. The management strategy to counter pathogens involves application of chemical pesticides, but this increases the cost of production and is also associated with human health and environmental hazards [1]. Improving host genetic resistance through development of disease resistance cultivars is the most sustainable and effective management strategy. Genome editing can improve disease resistance in commercial or elite breeding potato cultivars with less effort, time, enhanced precision and efficiency [2]. It is important to understand the molecular mechanisms behind the resistance and susceptibility to pathogen attack for genome editing in potato.

Upon pathogen detection, plants immediately activate programmed cell death (PCD), initially in the form of hypersensitive response (HR), which is termed HR-PCD [3]. HR is a localized plant cell death at the infection point to restrict the food supply to the pathogen, thus limiting its spread to the point of infection. True apoptosis is absent in plants, unlike in animals, but apoptotic-like PCD (AL-PCD), which is morphologically and biochemically similar to apoptosis in animals, has been reported [4]. AL-PCD is induced later than HR-PCD, but it is still a rapid process, initiating and often ending within 6 h [5]. The DNA ladders, a characteristic feature of AL-PCD, appear in 6 hpi of pathogen detection [6]. Though both HR-PCD and AL-PCD are characterized by cytoplasmic shrinkage, mitochondrial swelling, cytochrome c release, chloroplast disruption, chromatin condensation and DNA fragmentation, they differ in the presence of plasma membrane blebbing and a characteristic DNA fragmentation [7]. Both HR and AL-PCD are

detrimental to biotrophic pathogens as PCD limits its access to living cells and cuts off the nutrient source. However, hemibiotrophic and necrotrophic pathogens actively promote PCD in their host cells and utilize it to derive nutrients from the dead cells. Delaying or preventing AL-PCD can stop cell death, limiting the nutrient source for pathogens, resulting in enhanced plant resistance.

For pathogen detection, the pathogen-associated calcium ion (Ca^{2+}) influx signals are perceived by calcium sensors such as calmodulin (CaM), CaM-like proteins, Ca^{2+} -dependent protein kinases (CDPKs) and Ca^{2+}- and Ca^{2+}/CaM-independent protein kinase (CCaMK) and calcineurin B-like proteins (CBLs) [8]. The pathogen-associated Ca^{2+} influx is perceived and transported to the nucleus by the sarcoplasmic/endoplasmic histidine-rich Ca^{2+} -binding protein (HRC) [9]. HRC, initially identified in fusarium head blight (FHB) resistant wheat near isogenic lines (NIL), seems to play an important role in Ca^{2+} mediated defense response [10]. Mutation in HRC, both naturally and through gene editing, leads to disease resistance; FHB resistance in wheat and blight resistance in potato. In wheat, *TaHRC* was found to be mutated in FHB resistant lines, while in potato, *StHRC* was silenced, where both lead to high resistance against blight diseases through suppression of AL-PCD [11-13].

Metacaspases mediate PCD in plants during development, and during abiotic and biotic stresses resistance and play important roles during AL-PCD [14, 15]. Two types of metacaspases are found in plants, type I metacaspase, which contains an N-terminal prodomain a subunit p20, and type II metacaspases, which contains a linker joining two subunits, p20 and p10 [16]. Changes in Ca^{2+} concentration affect the activity of metacaspases and endonucleases. Upon increased Ca^{2+} influx, metacaspases undergo conformational changes and is activated by multiple cleavage in the linker region of type II metacaspases [17]. *AtMC2d*, the most abundant type II metacaspases in Arabidopsis, exhibits Ca^{2+} dependency for activation [18]. Metacaspases then processes substates

such as Propep1, producing Pep1 elicitors, which trigger downstream immune response and signal nearby cells [19]. Among the 9 metacaspases in potato, *StMC7*, showing significant similarity to *AtMC4* in arabidopsis, is constitutively highly expressed in most plant tissues [20]. Silencing of *StHRC* also reduced the expression of *StMC7* and increased the disease resistance to *Phytophthora infestans* and *Alternaria solani* [13]. Accordingly, it was hypothesized that the silencing of *StMC7* may also lead to the suppression of AL-PCD, thus inhibiting the infection process by hemibiotrophic and necrotrophic pathogens, due to suppression of their nutritional source, leading to increased plant resistance.

Here, we report *Solanum tuberosum* metacaspase 7 (*StMC7*) as a hemibiotrophic pathogen susceptibility gene, based on plant-pathogen interactions with the hemibiotrophic pathogen *Phytophthora infestans*. CRISPR/Cas9 mediated knockout of *StMC7* in Russet Burbank potato showed reduction in gene expression, disease severity and pathogen biomass ~~for the pathogen~~. Disease severity for the necrotrophic pathogen *Alternaria solani* was also reduced in *Stmc7* mutants. Based on these findings, we report that silencing of the functional *StMC7* leads to enhanced disease resistance to late blight and may also provide multiple disease resistance in potato.

Materials and methods

Plant production

Tissue cultured plantlets of the susceptible potato genotype Russet Burbank (RB) was obtained from the New Brunswick Plant Propagation Center (Potato Research Centre, Agriculture and Agri-Food Canada, New Brunswick, Canada). The tissue culture plantlets were further multiplied in the lab and then grown in the greenhouse in pots with Fafard AGRO MIX® G6 under greenhouse

condition (20 ± 3°C temperature, 70 ± 10% relative humidity, 16 hr photoperiod, 1500 µmol/m^2/s light intensity). The tissue cultured plantlets were grown in sterile half strength M516 media, supplemented with 3% sucrose and 2g/L Phytagel™. Plantlets were incubated in a Percival growth chamber at 22°C with 16 h light cycle. Internodal segments and leaf segments from 3-week-old sterile plants were used for *Agrobacterium*-mediated transformation.

DNA extraction and *StMC7* sequencing

DNA was extracted from RB potato seedlings using a modified CTAB method [21]. The full gene sequence of *StMC7* covering both exons was amplified with specific primers using a high fidelity Q5 Polymerase (New England Biolabs, Ipswich, MA, USA) (Table 1). The amplified PCR product was purified using BioBasic PCR Clean Up (Bio Basic INC., Markham, ON, CA). The purified PCR product was cloned into pGEMT-Easy plasmid (Promega Corp., Madison, WI, USA) using T4 DNA Ligase (NEB, MA, USA). The final plasmid was transformed into competent *E. coli* DH5α cells (New England Biolabs, Ipswich, MA, USA) and sequenced using M13-F and R primers by Sanger sequencing (Institute of Integrative Biology and Systems (IBIS), Laval University) sequences were translated with Expasy (https://www.expasy.org/) to obtain protein sequence [22]. The protein structure of *StMC7* was predicted using RoseTTAFold [23].

Table 1. Primers used for gRNA synthesis, amplicon sequencing, pathogen biomass quantification, qRT-PCR.

Gene name	Primer sequence
sgRNA-1	F: 5’- GATTGAATATACGTAAAGCTTTAT-3’<br>R: 5’-AAACATAAAGCTTTACGTATATTC-3’
sgRNA-2	F: 5’- GATTGAGCTATGCGAAACCAGCCA-3’<br>R: 5’-AAACTGGCTGGTTTCGCATAGCTC-3
sgRNA-3	F: 5’- GATTGCAGATGCCACCCCTGCAGG-3’<br>R: 5’-AAACCCTGCAGGGGTGGCATCTGC-3’

StMC7-gRNA-1	F: 5’- GCGAAAAAGGCAGTGTTAATTGG-3’<br>R: 5’- CCCCCAACAAATTCAACAAACAC-3’
StMC7-gRNA-2-3	F: 5’- TGGGAAACTTAGGCCAACAC-3’<br>R: 5’- AGGCATAGCACAGCAGAATC-3’
StMC7-amplicon	F:5’-<br>ACACTCTTTCCCTACACGACGCTCTTCCGATCTGCGAAAAAGG<br>CAGTGTTAATTGG-3’<br>F:5’-<br>GTGACTGGAGTTCAGACGTGTGCTCTTCCGATCTCCCCCAACA<br>AATTCAACAAACAC-3’
StEf1-α	F: 5’- ATTGGAAACGGATATGCTCCA -3’<br>R: 5’- TCCTTACCTGAACGCCTGTCA -3’
Stβ-tubulin	F: 5’- ATGTTCAGGCGCAAGGCTT-3’<br>R: 5’- TCTGCAACCGGGTCATTCAT-3’
O-8(Phytophthora infestans)	F: 5’- TGGGAAACTTAGGCCAACAC-3’<br>R: 5’- TAACCGACCAAGTAGT AAA -3’
StMC7-RT	F: 5’- GCCTTCTCGAGTAGCTGTTGA -3’<br>R: 5’- TCACATGATGGAGAATGGTT-3’

Vector construction and *Agrobacterium*-mediated transformation

Three guide RNAs (gRNAs) were designed based on the *StMC7* gene sequence, targeting both exons, one targeting the first exon and two targeting the second exon, using CRISPR-P 2.0 (http://crispr.hzau.edu.cn/CRISPR2/) [24]. The three gRNAs were individually cloned into three separate pDIRECT21A vectors through Golden Gate Assembly using AarI restriction enzyme [25]. The final vectors were transformed into *E. coli* DH5α competent cells and sequenced (IBIS, Laval University). The clones with successfully inserted gRNA were transformed using the standard freeze-thaw method into *Agrobacterium tumefaciens* (GV3101) for explant transformation.

Transformation was carried out according to Duan et al. (2012) with slight modifications [26]. Briefly, internodes and leaves were sterilized using 70% ethanol for 30s followed by 50% NaOCl solution for 10 min for internodes and 5 min for leaves. The explants were subjected to a mix of

three *Agrobacterium* suspension cultures harboring the three different constructs in pDIRECT_21A construct. The explants were transferred to co-cultivation media (1/10 MS salts, 3 % sucrose, pH 5.7, 6g/L agar) at 22ºC for 2 days and then transferred to callus induction media (CIM) (4.3 g/L MS salts, 2.5 mg/L zeatin, 0.1 mg/L naphthalene acetic acid (NAA), 3 % sucrose. 6 g/L agar) with timentin (150 mg/L) and hygromycin (50 mg/L) and incubated at 22ºC. After 1-month, growing calli were transferred to shoot induction media (SIM) (4.3 g/L MS media, 2.5 mg/L zeatin, 0.3 mg/L gibberellic acid, 6 g/L sugar, 3 % sucrose) with timentin (150 mg/L) and hygromycin (50 mg/L). Calli with emerging shoots were transferred to MS media supplemented with 3% sucrose, 6 g/L agar, supplemented with previous dose of timentin and hygromycin.

Detection of putative CRISPR-Cas9 potato mutants

A 526-bp surrounding the *StMC7* gRNA1 region was amplified using Phusion Green Hot Start II High-Fidelity PCD Master Mix (ThermoFisher Scientific, MA, USA) and purified using the BioBasic PCR Purification Kit (Biobasic, ON, CA). The purified sample was subjected to restriction digestion assay using HindIII, which cut at a unique site within the gRNA 1, as per the manufacturer's protocol. The purified sample was sent for sequencing by Illumina NextSeq PE300-500K at Genome Quebec, Montreal. Results from amplicon sequencing were analyzed using CRISPResso2 [27].

Disease severity and pathogen biomass assay

Phytophthora infestans isolate US-8, A2 mating type, a highly virulent, aggressive strain, (received from Dr. H. Platt, AAFC, Charlestown, PEI, CA) was maintained on potato dextrose agar (PDA). Spores were produced by inoculating a thin potato tuber slice with *P. infestans* and incubating the sealed and moist Petri dish in 18ºC for sporulation. Sporangia were harvested and spore

concentration was adjusted to 10^5 sporangia per ml. *Alternaria solani* (obtained from Dr. A. Dionne, MAPAQ, QC) was maintained on PDA. For spore production, the plate was incubated at room temperature with 12 hr light and dark photoperiod.

The experiment for pathogen biomass and disease severity was conducted in a Randomized Complete Block Design (RCBD) with two genotypes (wild RB and *StMC7* mutants), two inoculations (mock and pathogen), and three temporal replicates. Each experimental unit consisted of five pots with two plants and ten leaves inoculated in each pot. Young leaves of 5-6 weeks old plants were point inoculated on the either side of midrib on the lower surface with 10 µl sporangial suspension. The plants were covered with plastic bags upon inoculation for 72 h and 48 h for *P. infestans* and *A. solani*, respectively. Disease severity was quantified by measuring the lesion diameter (in mm) at 3 days interval until 9 post-inoculation (dpi) and calculating the area under the disease progress curve (AUDPC). Relative biomass for *P. infestans* from infected samples was quantified based on quantitative PCR (qPCR) to determine the biomass of the pathogens in infected samples. Genomic DNA was isolated from the infected leaves 6 dpi using the CTAB method. Specific primers for *P. infestans*, as well as against the potato genome was used in qPCR performed using a Luna Universal qPCR Mastermix (NEB, MA, USA) using Mic qPCR Cycler (Bio Molecular Systems, Queensland, Australia) (Table 1) [28]. Statistical analysis was conducted using SPSS (IBM SPSS Statistics 29.0).

RNA extraction and gene expression by RT-qPCR.

NCBI BLAST was used to design RT-qPCR primers [29]. RNA was extracted from the leaves of RB genotype and *Stmc7* knockout plants 6 days post inoculation (dpi) following *P. infestans* and ~~maock~~ (water) inoculation using RNeasy Plant Mini Kit (Qiagen, Venlo, Netherlands). cDNA was synthesized using Maxima H Minus First Strand cDNA Synthesis Kit (ThermoFisher Scientific,

MA, USA). qPCR was conducted with Luna Universal qPCR Mastermix (NEB, MA, USA). Two genes, elongation factor alpha (*StEf1α)* and *tubulin* (*Sttubulin*) were used as reference genes. The relative gene expression levels were analyzed based on delta-delta C_T (cycle threshold) method [30]. All the gene expression values are presented as gene expression ratio, obtained through comparison with RB mock inoculation.

Results

Characterization of *StMC7*

The *StMC7* gene was found to be 3061 bp, with two exons, 346 bp and 905 bp (Fig 1A). The gene sequence has been submitted to NCBI GenBank (Accession number PQ811591). Protein sequence analysis revealed the presence of a peptidase C14 caspase domain, supporting the role of *StMC7* as a metacaspase (Fig 1B). Upon comparison with arabidopsis *AtMC4* sequence, the exact positions for conserved caspase-like catalytic domains such as the p-20 subunit (20 kDa), p-10 subunit (10 kDa) and linker between them were identified. The catalytic histidine was found within the sequence HYSGHG and the catalytic cysteine was found within DSCHS, similar to *AtMC4* (Fig 1B) [31]. The protein structure of *StMC7* was predicted using RoseTTAFold, revealing its catalytically inactive state (Fig 1C).

Fig 1 Characterization of the potato metacaspase *StMC7* **(A)** gene structure showing the two coding sequence regions, corresponding to the p20 and p10 domain associated with type II metacaspases. **(B)** StMC7 protein structure with the domains, p20 and p10 region, linker region and the conserved catalytic histidine and cysteine within the p20 region. **(C)** Predicted protein structure of StMC7 obtained using RoseTTAFold.

StMC7 knockout based on CRISPR-Cas9

Plants with InDel mutations were generated targeting the *StMC7* exon 1 through *Agrobacterium*-mediated transformation of the CRISPR-Cas9 construct (Fig 2) in the late blight susceptible cultivar Russet Burbank (RB). Transformed internodes and leaves successfully produced callus under the selection of hygromycin, resulting in putative transgenic potato plants (Fig 3). Eight plants were obtained after hygromycin selection through every stage of tissue culture. Restriction digestion assay showed InDel mutations in 6 plants at gRNA 1 (Fig 4A). No mutations were observed upon Sanger sequencing for gRNA 2 and 3. Amplicon-sequencing followed by CRISPResso2 analysis determined five heterozygous mutants in exon 1 of *StMC7* (Fig 4B).

Figure 2 CRISPR design and construct preparation. gRNA was selected from the Exon 1 of *StMC7* and introduced into the pDIRECT_21A vector using Golden Gate Assembly at the AarI position of the vector to prepare CRISPR construct.

Figure 3 *Stmc7* mutant regeneration from callus regeneration of internodes and leaves of RB potato using *Agrobacterium*-mediated transformation for CRISPR construct delivery. **(A)** Regenerating plantlets after *Agrobacterium*-mediated transformation of infected leaves and internodal segments. **(B)** Transgenic seedlings growing in shoot regeneration media **(C)** *Stmc7* mutant.

Figure 4 CRISPR-Cas9-mediated mutation analysis of *Stmc7* mutants. **(A)** HindIII digestion assay to identify *Stmc7* mutants. L: 1 kb plus ladder, RB: Positive Control, 1-8: *Stmc7* transgenic samples. Wild type RB (lane RB) or non-edited samples (lane 6,8) gives two bands. **(B)** Percentage of total modified reads for each *Stmc7* mutant line. The value at the top of the bar represents the exact percentage of modified reads. **(C)** Indels detected in *Stmc7* mutants by targeted amplicon

sequencing mapped to wild type sequence. The percentage of reads for each sequence and no. of reads with that sequence are shown on the right. Sequences below 2% are not shown. The asterisk (*) indicates non-edited reads. Blue vertical dash line indicates predicted cleavage position, black horizontal dash indicates base deletion.

Stmc7 reduced disease severity and pathogen biomass in Russet Burbank potato.

Disease severity was assessed among *Stmc7*, and *Sthrc* mutants, and RB non-edited as control. Though circular lesions began to appear in all samples from 3 dpi for both early and late blight diseases, the lesions in RB control were much larger than *Stmc7* and *Sthrc*. The area under disease progress curve (AUDPC) was seven-fold lower in *Stmc7* mutants (36.25) as compared to RB (249) and two-fold lower than *Sthrc* mutants (77.25). *StMC7* was upregulated in pathogen-infected RB wildtype plants compared to the *Stmc7* mutants for *P. infestans* inoculated leaves (Fig 5D). Pathogen biomass in the infected leaves with *P. infestans* specific primers quantified based on qPCR at 6 dpi were higher in RB compared to the *Stmc7* mutants (Fig 5), which reflects lower *P. infestans* levels in *Stmc7* mutants compared to the RB wildtype (Fig 5C). In *A. solani* infected leaves, the AUDPC, calculated based on lesion diameter, was twice as low in *Stmc7* mutants (19.5) compared to RB (43.5). *StMC7* gene expression was lower in *Stmc7* mutants for *P. infestans* (3.5-fold) inoculated leaves compared to pathogen inoculated RB plants (Fig. 5D).

Figure 5 Russet Burbank (RB) potato leaves, with functional and mutated metacaspase *Stmc7*, inoculated with *Phytophthora infestans*. **(A)** Disease progression based on the lesion diameter (mm) at 3-, 6- and 9-days post inoculation (dpi) **(B)** Late blight symptoms showing highly significant differences between RB and *Stmc7* mutants ($p<0.001$), significant differences between *StHRC* and *StMC7* mutants ($p = 0.02$). **(C)** Pathogen biomass quantification at 6 days

post-inoculation, quantified as relative P. infestans specific (*O-8*) gene expression **(D)** Relative gene expression of *StMC7* in RB and *Stmc7* mutants compared to the reference genes *StEf1* and *tubulin*, relative to wildtype mock inoculation, following *Phytophthora infestans* inoculation. Significance determined by Student's t-test, P value: **p<0.01, *p<0.05, or multiple comparison test (Tukey's HSD), significant differences shown by lowercase letters ($p < 0.05$). Error bars represent mean standard error (SEM) and asterisks denote values significantly different to wild type ($p < 0.05$).

Figure 6 Russet Burbank potato leaves, with functional and mutated metacaspase *Stmc7*, infected with *Alternaria solani*. **(a)** Disease progress based on the lesion diameter (mm) at 3-, 6- and 9-days post inoculation **(b)** Early blight symptoms showing differences between RB and *Stmc7* mutants.

Discussion

Programmed cell death (PCD) is an important defense strategy against biotrophic pathogens in plants. Hemibiotrophic oomycetes, such as *Phytophthora infestans* have a short, sometimes cryptic, biotrophic phase before switching to necrotrophic phase [32]. These pathogens induce effectors to elicit effector triggered immunity (ETI) and several elicitors or molecular patterns to elicit pattern triggered immunity (PTI) in plants, leading to HR-PCD. *Phytophthora infestans* produces fatty acids, such as Arachidonic acid, apoplastic effectors, such as necrotizing toxins Nep1-like proteins (NLPs), PcF-like (*Phytophthora cactorum* in *Fragaria*-like) small cysteine-rich proteins (SCRs)), and cytoplasmic effectors, such as CRN (crinkling, necrosis), etc. to induce AL-PCD [33, 34]. Necrotrophic pathogen such as *Alternaria* spp. produces non-host specific toxins such as alternariol, zinniol, tentoxin and host-specific toxin families to elicit host defense response suppression and stimulate cell death [35]. This induction of cell death through

PCD in plants is important in increasing food supply for necrotrophic and hemibiotrophic pathogens [36]. Suppressing cell death by silencing host PCD pathway genes can confer resistance to such pathogens [13].

In this study, a key PCD pathway gene, metacaspase 7 (*StMC7*) was silenced using CRISPR gene editing technology, generating *Stmc7* mutants with InDel mutations in exon 1. The reduction in the AUDPC for both early and late blights possibly indicate successful suppression of pathogen attack in the mutants. For *P. infestans*, there were lower pathogen growth in *Stmc7* mutants compared to control RB plants (Fig 5C). For *A. solani*, disease progression was twice as slow in *Stmc7* mutants compared to the wild type. Further experiments under field conditions are necessary to confirm the *Stmc7* mutants' response to *P. infestans* and *A. solani* infection.

Metacaspases such as *StMC7* have been involved in plant pathogen resistance in arabidopsis, tomato, wheat, and pepper [16]. In arabidopsis, mutations in type 2 metacaspases, *AtMC2* to *AtMC6* resulted in reduced susceptibility to *Botrytis cinera* and *B. tulipae* [37]. In tomato, *LeMC1* was rapidly induced when infected by *B. cinera* [38]. In wheat, the expression of *TaMC4* was significantly upregulated when challenged with *Puccinia striiformis* f.sp. *tritici*. Virus-induced gene silencing (VIGS) of *TaMC4* resulted in limited fungal growth [39]. Another metacaspase, *TaMC1* was significantly upregulated upon infection by *Puccinia striiformis* and increased disease resistance upon knockdown of *TaMC1* expression through VIGS [40]. VIGS of metacaspase 9 in pepper (*CaMC9*) lead to delayed cell death symptoms and reduced cell death induced by *Xanthomonas campestris* pv. *vesicatoria* while the overexpression of *CaMC9* enhanced cell death and increased susceptibility to *Pseudomonas syringae* pv. *tabaci* [41]. In potato, *StMC7* was significantly downregulated upon silencing histidine-rich calcium-binding protein coding gene (*StHRC*) indicating its prominent role in inducing AL-PCD [13].

Though PCD is induced during plant-pathogen interaction, the exact pathway of PCD induction is not clear. An essential condition for metacaspases activation is an increase in Ca^{2+}. A model on the pathway of AL-PCD induction in potato has been proposed by silencing *StHRC* [13]. In general, an influx of intracellular Ca^{2+} following fungal pathogen is sensed by various Ca^{2+} influx protein sensors responsible for maintaining cytosolic Ca^{2+} homeostasis [42]. Ca^{2+} -transporters such as Ca^{2+} -ATPases, two-pore Ca^{2+} channels and cyclic nucleotide gated channels (CNGCs), transport Ca^{2+} out of cytosol, across various cellular membranes [43]. When Ca^{2+} ion concentration increases in mitochondria, cytochrome c is released via the formation of permeability transition (PT) pore or through the large cytochrome c-conducting channel formed by voltage-dependent anion channel (VDAC), increasing the reactive oxygen species (ROS) [44]. Ca^{2+} - binding proteins such as *StHRC* regulate the downstream response processes [45]. HRC transports the Ca^{2+} into nucleus, where the Ca^{2+} triggers Ca^{2+}- dependent endonucleases such as *StCaN2*, to degrade DNA [13, 46]. A higher concentration of Ca^{2+} triggers the activation of *StMC7* in the cytosol through multiple cleavage at 6 sites in the linker region releasing the linker region and making the active site available for substrate processing [17]. Structures such as apoptosome have been reported to activate caspases and execute AL-PCD in animals [47]. Cryo-electron microscopy may elucidate if similar structures may form before AL-PCD in potato. Silencing genes involved in apoptosome, and other candidate genes involved in AL-PCD, endonucleases such as *StCaN2*, could also reduce the DNA fragmentation, induction of AL-PCD and provide further multiple disease resistance.

The mechanism of resistance through HR-PCD is the death of cells that limit food supply stopping the growth of a biotrophic pathogen. However, in AL-PCD the mechanism of resistance is quite complex. The dead cells increase food supply, increasing the growth of hemibiotrophic and

necrotrophic pathogens. Whereas the silencing of AL-PCD leads to reduced disease severity, and the reduction is considered not to be due to resistance genes. However, the induction of AL-PCD increases pathogen biomass, which produces more toxins. These toxins may also suppress the expression of genes responsible for resistance, as observed in wheat infected with *Fusarium graminearum* which produces deoxynivalenol that inhibits the eukaryotic protein translation including enzymes catalyzing several RR metabolites [48]. Thus, the silencing of AL-PCD not only reduces the susceptibility but also increases the resistance by enabling the expression of resistance genes.

Conclusion

Potato cultivars with multiple disease resistance are the desired outcome for all potato breeding programs. PCD is an important pathway in plant-pathogen interactions, which when suppressed may increase resistance to multiple pathogens. Upon pathogen- or damage-associated molecular pattern recognition, the plant activates the HR-PCD pathway. Apoptotic-like PCD has also been observed in plants, which involves plasma membrane blebbing and characteristic DNA fragmentation. AL-PCD generates nutrient sources for hemibiotrophic and necrotrophic pathogens. By silencing one of the important gene that mediate AL-PCD, a metacaspase *StMC7*, we have shown increased resistance against important potato pathogen, *Phythophthora infestans*, causing late blight and possible resistance to *Alternaria solani*, causing early blight of potato. Further experiments with other hemibiotrophic and necrotrophic pathogens on *StMC7* mutants should prove this to be a common multiple disease resistance mechanism in plants.

REFERENCES

1. Ivanov AA, Ukladov EO, Golubeva TS. Phytophthora infestans: An Overview of Methods and Attempts to Combat Late Blight. Journal of Fungi. 2021;7(12):1071. PubMed PMID: doi:10.3390/jof7121071.
2. Movahedi A, Aghaei-Dargiri S, Li H, Zhuge Q, Sun W. CRISPR Variants for Gene Editing in Plants: Biosafety Risks and Future Directions. Int J Mol Sci. 2023;24(22). Epub 20231113. doi: 10.3390/ijms242216241. PubMed PMID: 38003431; PubMed Central PMCID: PMCPMC10671001.
3. Camagna M, Takemoto D. Hypersensitive Response in Plants. eLS2018. p. 1-7.
4. Reape TJ, McCabe PF. Apoptotic-like programmed cell death in plants. New Phytologist. 2008;180(1):13-26. doi: 10.1111/j.1469-8137.2008.02549.x
5. Dickman M, Williams B, Li Y, de Figueiredo P, Wolpert T. Reassessing apoptosis in plants. Nature Plants. 2017;3(10):773-9. doi: 10.1038/s41477-017-0020-x.
6. Balk J, Chew SK, Leaver CJ, McCabe PF. The intermembrane space of plant mitochondria contains a DNase activity that may be involved in programmed cell death. Plant J. 2003;34(5):573-83. doi: 10.1046/j.1365-313x.2003.01748.x. PubMed PMID: 12787240.
7. Coll NS, Epple P, Dangl JL. Programmed cell death in the plant immune system. Cell Death & Differentiation. 2011;18:1247-56. doi: 10.1038/cdd.2011.37.
8. Galon Y, Finkler A, Fromm H. Calcium-Regulated Transcription in Plants. Molecular Plant. 2010;3:653-69. doi: 10.1093/mp/ssq019.
9. Chen H, Su Z, Tian B, Hao G, Trick HN, Bai G. TaHRC suppresses the calcium-mediated immune response and triggers wheat Fusarium head blight susceptibility. Plant Physiology. 2022;190:1566-9. doi: 10.1093/plphys/kiac352.
10. Gunnaiah R. Functional characterization of wheat, fusarium head blight resistance (QTL) Fhb1 based on non-target metabolomics and proteomics. Ph.D. Dissertation, McGill University; 2013. Available from: https://escholarship.mcgill.ca/concern/theses/k643b439k
11. Su Z, Jin S, Zhang D, Bai G. Development and validation of diagnostic markers for Fhb1 region, a major QTL for Fusarium head blight resistance in wheat. Theoretical and Applied Genetics. 2018;131:2371-80. doi: 10.1007/s00122-018-3159-6.
12. Li G, Zhou J, Jia H, Gao Z, Fan M, Luo Y, et al. Mutation of a histidine-rich calcium-binding-protein gene in wheat confers resistance to Fusarium head blight. Nature Genetics. 2019;51:1106-12. doi: 10.1038/s41588-019-0426-7.

13. Kushalappa AC, Hegde NG, Gunnaiah R, Sathe A, Yogendra KN, Ajjamada L. Apoptotic-like PCD inducing HRC gene when silenced enhances multiple disease resistance in plants. Scientific Reports. 2022;12:20402. doi: 10.1038/s41598-022-24831-0.

14. Suarez MF, Filonova LH, Smertenko A, Savenkov EI, Clapham DH, von Arnold S, et al. Metacaspase-dependent programmed cell death is essential for plant embryogenesis. Current Biology. 2004;14:R339-R40. doi: 10.1016/j.cub.2004.04.019.

15. Watanabe N, Lam E. Calcium-dependent Activation and Autolysis of Arabidopsis metacaspase 2d. Journal of Biological Chemistry. 2011;286:10027-40. doi: 10.1074/jbc.M110.194340.

16. Garcia N, Kalicharan RE, Kinch L, Fernandez J. Regulating Death and Disease: Exploring the Roles of Metacaspases in Plants and Fungi. Int J Mol Sci. 2022;24. Epub 20221224. doi: 10.3390/ijms24010312. PubMed PMID: 36613753; PubMed Central PMCID: PMCPMC9820594.

17. Zhu P, Yu X-H, Wang C, Zhang Q, Liu W, McSweeney S, et al. Structural basis for Ca2+-dependent activation of a plant metacaspase. Nature Communications. 2020;11:2249. doi: 10.1038/s41467-020-15830-8.

18. Watanabe N, Lam E. Arabidopsis metacaspase 2d is a positive mediator of cell death induced during biotic and abiotic stresses. The Plant Journal. 2011;66:969-82. doi: 0.1111/j.1365-313X.2011.04554.x

19. Hander T, Fernández-Fernández ÁD, Kumpf RP, Willems P, Schatowitz H, Rombaut D, et al. Damage on plants activates Ca2+-dependent metacaspases for release of immunomodulatory peptides. Science. 2019;363(6433):eaar7486. doi: 10.1126/science.aar7486.

20. Dubey N, Trivedi M, Varsani S, Vyas V, Farsodia M, Singh SK. Genome-wide characterization, molecular evolution and expression profiling of the metacaspases in potato (*Solanum tuberosum* L.). Heliyon. 2019;5. doi: 10.1016/j.heliyon.2019.e01162.

21. Brauer EK, Balcerzak M, Rocheleau H, Leung W, Schernthaner J, Subramaniam R, et al. Genome Editing of a Deoxynivalenol-Induced Transcription Factor Confers Resistance to *Fusarium graminearum* in Wheat. Molecular Plant-Microbe Interactions®. 2020;33(3):553-60. doi: 10.1094/mpmi-11-19-0332-r. PubMed PMID: 31790345.

22. Gasteiger E, Gattiker A, Hoogland C, Ivanyi I, Appel RD, Bairoch A. ExPASy: The prediction proteomics server for in-depth protein knowledge and analysis. Nucleic Acids Res.

2003;31:3784-8. doi: 10.1093/nar/gkg563. PubMed PMID: 12824418; PubMed Central PMCID: PMCPMC168970.

23. Baek M, DiMaio F, Anishchenko I, Dauparas J, Ovchinnikov S, Lee GR, et al. Accurate prediction of protein structures and interactions using a three-track neural network. Science. 2021;373:871-6. doi: 10.1126/science.abj8754.

24. Liu H, Ding Y, Zhou Y, Jin W, Xie K, Chen L-L. CRISPR-P 2.0: An Improved CRISPR-Cas9 Tool for Genome Editing in Plants. Molecular Plant. 2017;10:530-2. doi: 10.1016/j.molp.2017.01.003.

25. Čermák T, Curtin SJ, Gil-Humanes J, Čegan R, Kono TJY, Konečná E, et al. A Multipurpose Toolkit to Enable Advanced Genome Engineering in Plants. Plant Cell. 2017;29:1196-217. Epub 20170518. doi: 10.1105/tpc.16.00922. PubMed PMID: 28522548; PubMed Central PMCID: PMCPMC5502448.

26. Duan H, Richael C, Rommens CM. Overexpression of the wild potato eIF4E-1 variant Eva1 elicits Potato virus Y resistance in plants silenced for native eIF4E-1. Transgenic Research. 2012;21(5):929-38. doi: 10.1007/s11248-011-9576-9.

27. Clement K, Rees H, Canver MC, Gehrke JM, Farouni R, Hsu JY, et al. CRISPResso2 provides accurate and rapid genome editing sequence analysis. Nature Biotechnology. 2019;37:224-6. doi: 10.1038/s41587-019-0032-3.

28. Hegde N, Joshi S, Soni N, Kushalappa AC. The caffeoyl-CoA O-methyltransferase gene SNP replacement in Russet Burbank potato variety enhances late blight resistance through cell wall reinforcement. Plant Cell Rep. 2021;40(1):237-54. Epub 20201103. doi: 10.1007/s00299-020-02629-6. PubMed PMID: 33141312.

29. Sayers EW, Bolton EE, Brister JR, Canese K, Chan J, Comeau DC, et al. Database resources of the national center for biotechnology information. Nucleic Acids Res. 2022;50(D1):D20-d6. doi: 10.1093/nar/gkab1112. PubMed PMID: 34850941; PubMed Central PMCID: PMCPMC8728269.

30. Livak KJ, Schmittgen TD. Analysis of relative gene expression data using real-time quantitative PCR and the 2(-Delta Delta C(T)) Method. Methods. 2001;25(4):402-8. doi: 10.1006/meth.2001.1262. PubMed PMID: 11846609.

31. Vercammen D, van de Cotte B, De Jaeger G, Eeckhout D, Casteels P, Vandepoele K, et al. Type II metacaspases Atmc4 and Atmc9 of Arabidopsis thaliana cleave substrates after arginine

and lysine. J Biol Chem. 2004;279(44):45329-36. Epub 20040823. doi: 10.1074/jbc.M406329200. PubMed PMID: 15326173.

32. Rajarammohan S. Redefining Plant-Necrotroph Interactions: The Thin Line Between Hemibiotrophs and Necrotrophs. Frontiers in Microbiology. 2021;12. doi: 10.3389/fmicb.2021.673518.

33. Haas BJ, Kamoun S, Zody MC, Jiang RHY, Handsaker RE, Cano LM, et al. Genome sequence and analysis of the Irish potato famine pathogen Phytophthora infestans. Nature. 2009;461:393-8. doi: 10.1038/nature08358.

34. Knight VI, Wang H, Lincoln JE, Lulai EC, Gilchrist DG, Bostock RM. Hydroperoxides of fatty acids induce programmed cell death in tomato protoplasts. Physiological and Molecular Plant Pathology. 2001;59:277-86. doi: 10.1006/pmpp.2001.0366.

35. Meena M, Gupta SK, Swapnil P, Zehra A, Dubey MK, Upadhyay RS. Alternaria Toxins: Potential Virulence Factors and Genes Related to Pathogenesis. Frontiers in Microbiology. 2017;8. doi: 10.3389/fmicb.2017.01451.

36. Ye C, Zheng S, Jiang D, Lu J, Huang Z, Liu Z, et al. Initiation and Execution of Programmed Cell Death and Regulation of Reactive Oxygen Species in Plants. Int J Mol Sci. 2021;22(23). Epub 20211130. doi: 10.3390/ijms222312942. PubMed PMID: 34884747; PubMed Central PMCID: PMCPMC8657872.

37. Van Baarlen P, Woltering EJ, Staats M, Van Kan JAL. Histochemical and genetic analysis of host and non-host interactions of Arabidopsis with three Botrytis species: an important role for cell death control. Molecular Plant Pathology. 2007;8:41-54. doi: 10.1111/j.1364-3703.2006.00367.x

38. Hoeberichts FA, ten Have A, Woltering EJ. A tomato metacaspase gene is upregulated during programmed cell death in *Botrytis cinerea*-infected leaves. Planta. 2003;217(3):517-22. doi: 10.1007/s00425-003-1049-9.

39. Wang X, Wang X, Feng H, Tang C, Bai P, Wei G, et al. TaMCA4, a novel wheat metacaspase gene functions in programmed cell death induced by the fungal pathogen *Puccinia striiformis* f. sp. *tritici*. Mol Plant Microbe Interact. 2012;25:755-64. doi: 10.1094/mpmi-11-11-0283-r. PubMed PMID: 22352715.

40. Hao Y, Wang X, Wang K, Li H, Duan X, Tang C, et al. TaMCA1, a regulator of cell death, is important for the interaction between wheat and Puccinia striiformis. Scientific Reports. 2016;6:26946. doi: 10.1038/srep26946.

41. Kim SM, Bae C, Oh SK, Choi D. A pepper (*Capsicum annuum* L.) metacaspase 9 (*Camc9*) plays a role in pathogen-induced cell death in plants. Mol Plant Pathol. 2013;14:557-66. Epub 20130325. doi: 10.1111/mpp.12027. PubMed PMID: 23522353; PubMed Central PMCID: PMCPMC6638822.

42. Köster P, DeFalco TA, Zipfel C. Ca(2+) signals in plant immunity. Embo j. 2022;41:e110741. Epub 20220513. doi: 10.15252/embj.2022110741. PubMed PMID: 35560235; PubMed Central PMCID: PMCPMC9194748.

43. Park C-J, Shin R. Calcium channels and transporters: Roles in response to biotic and abiotic stresses. Frontiers in Plant Science. 2022;13. doi: 10.3389/fpls.2022.964059.

44. Jones A. Does the plant mitochondrion integrate cellular stress and regulate programmed cell death? Trends Plant Sci. 2000;5:225-30. doi: 10.1016/s1360-1385(00)01605-8. PubMed PMID: 10785669.

45. Lee H-J, Seo PJ. Ca2+catalyzing Initial Responses to Environmental Stresses. Trends in Plant Science. 2021;26:849-70. doi: 10.1016/j.tplants.2021.02.007.

46. Sui W, Guo K, Li L, Liu S, Takano T, Zhang X. Arabidopsis Ca2+-dependent nuclease AtCaN2 plays a negative role in plant responses to salt stress. Plant Science. 2019;281:213-22. doi: 10.1016/j.plantsci.2018.12.007

47. Bao Q, Shi Y. Apoptosome: a platform for the activation of initiator caspases. Cell Death & Differentiation. 2007;14(1):56-65. doi: 10.1038/sj.cdd.4402028.

48. Gunnaiah R, Kushalappa AC. Metabolomics deciphers the host resistance mechanisms in wheat cultivar Sumai-3, against trichothecene producing and non-producing isolates of Fusarium graminearum. Plant Physiol Biochem. 2014;83:40-50. Epub 20140717. doi: 10.1016/j.plaphy.2014.07.002. PubMed PMID: 25084325.

Figure Legends

Fig 1. Characterization of the potato metacaspase *StMC7*.

Fig 2. CRISPR design and construct preparation.

Fig 3. Stmc7 mutant regeneration from callus regeneration of internodes and leaves of RB potato using *Agrobacterium*-mediated transformation for CRISPR construct delivery.

Fig 4. CRISPR-Cas9-mediated mutation analysis of *Stmc7* mutants.

Fig 5. Russet Burbank (RB) potato leaves, with functional and mutated metacaspase *Stmc7*, inoculated with *Phytophthora infestans*.

Fig 6. Russet Burbank potato leaves, with functional and mutated metacaspase *Stmc7*, infected with *Alternaria solani*.

Figure 1

Click here to access/download;Figure;pLOS-one-image-1.tiff

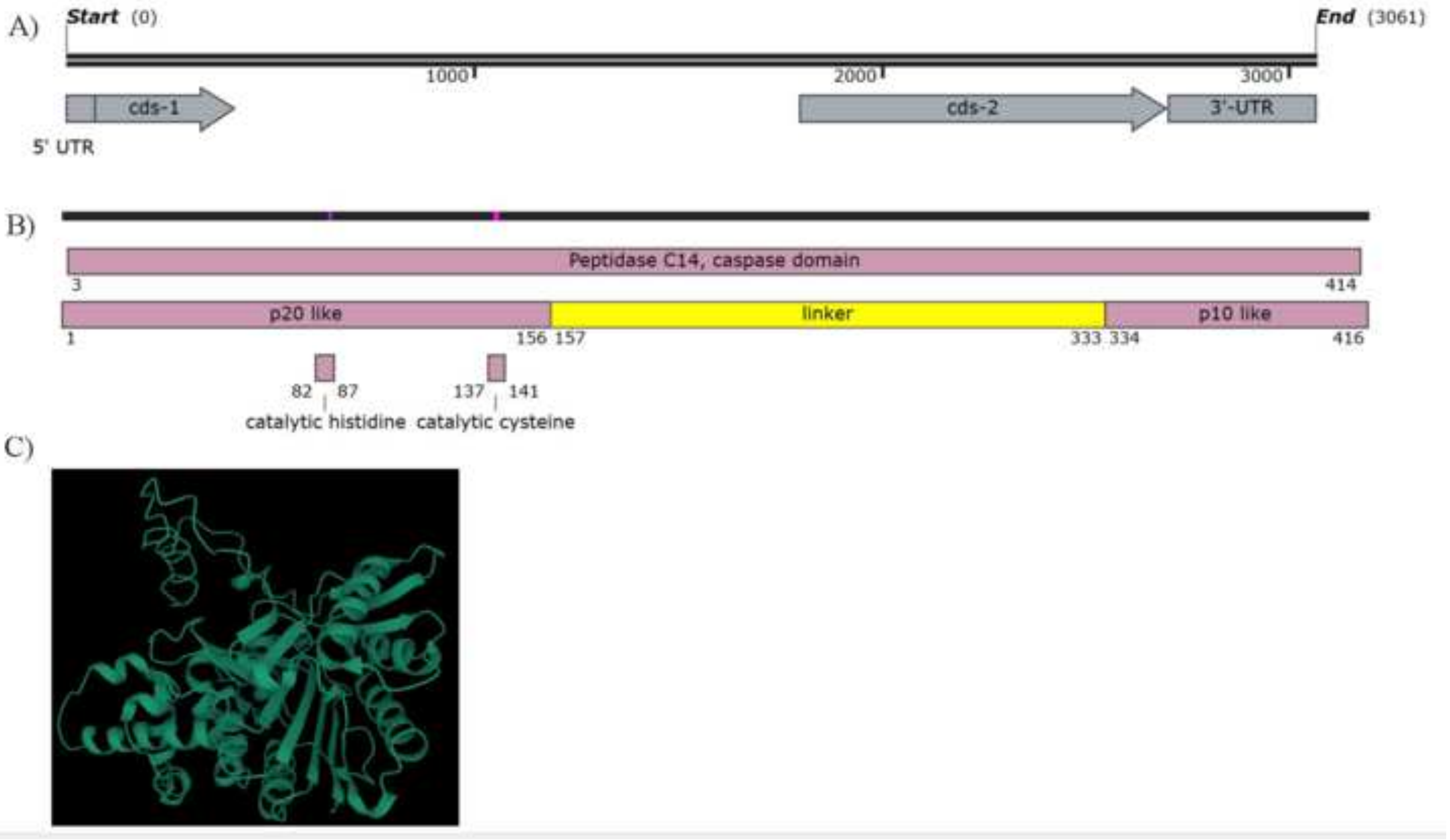

Figure 2 Click here to access/download;Figure;pLOS-one-image-2.tiff

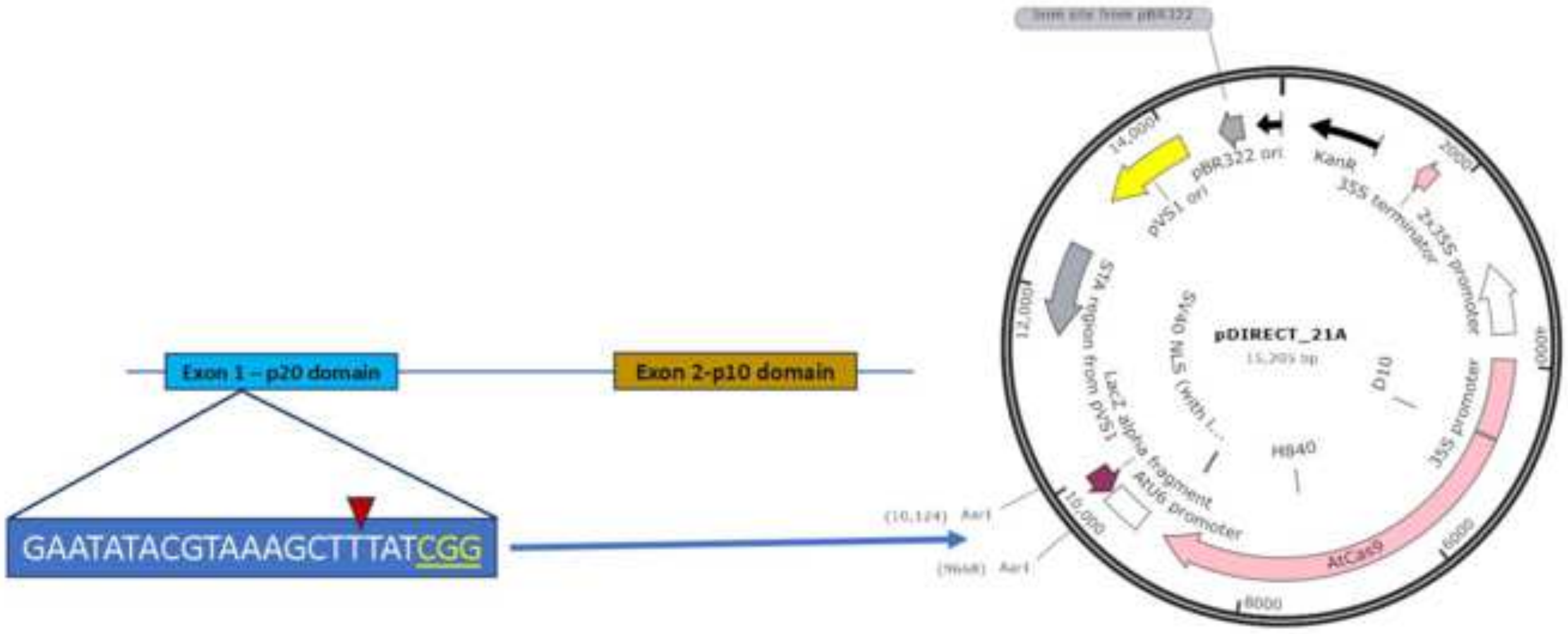

Figure 3

Click here to access/download;Figure;pLOS-one-image-3.tiff

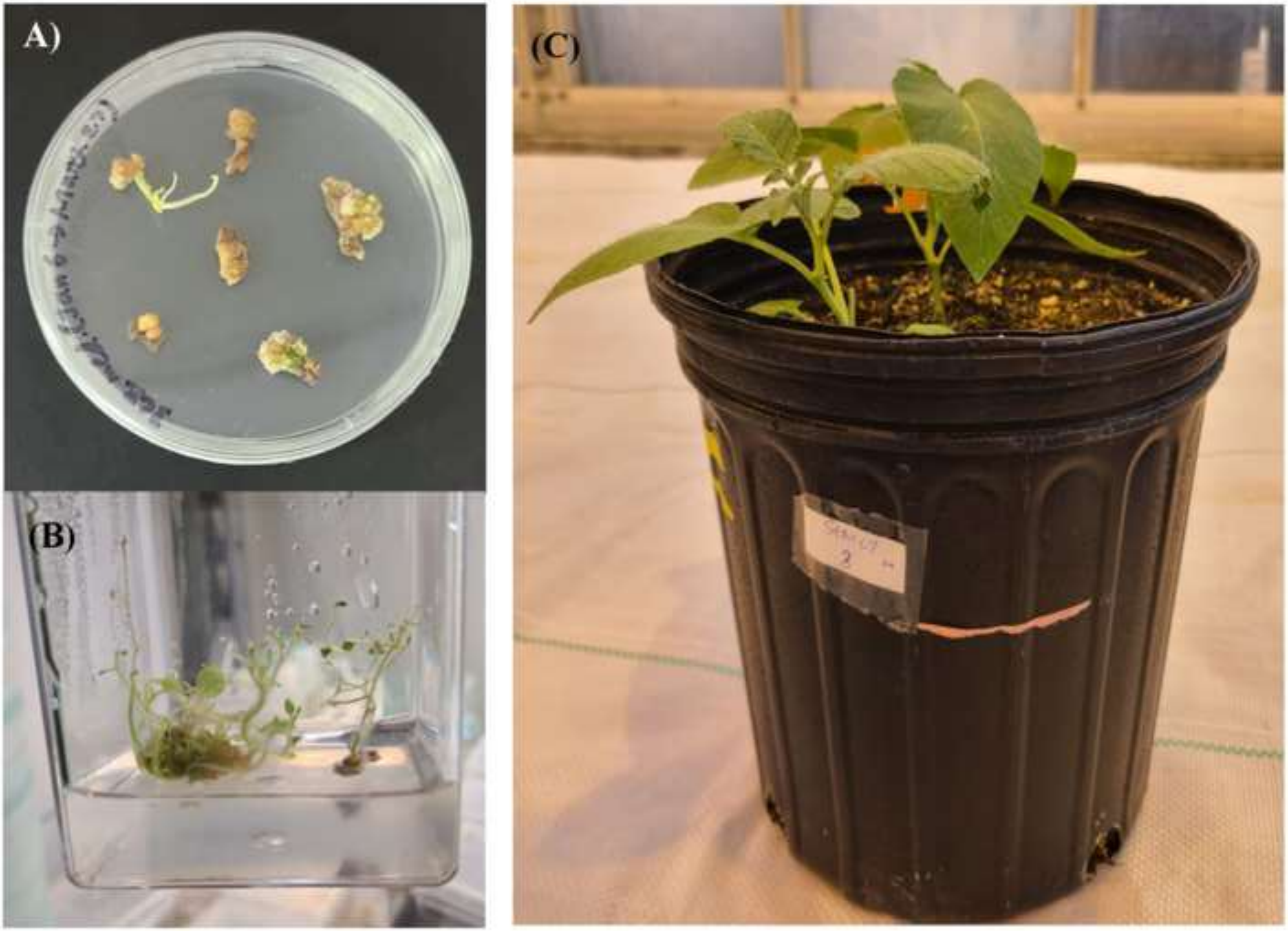

Figure 4

Click here to access/download;Figure;pLOS-one-image-4.tiff

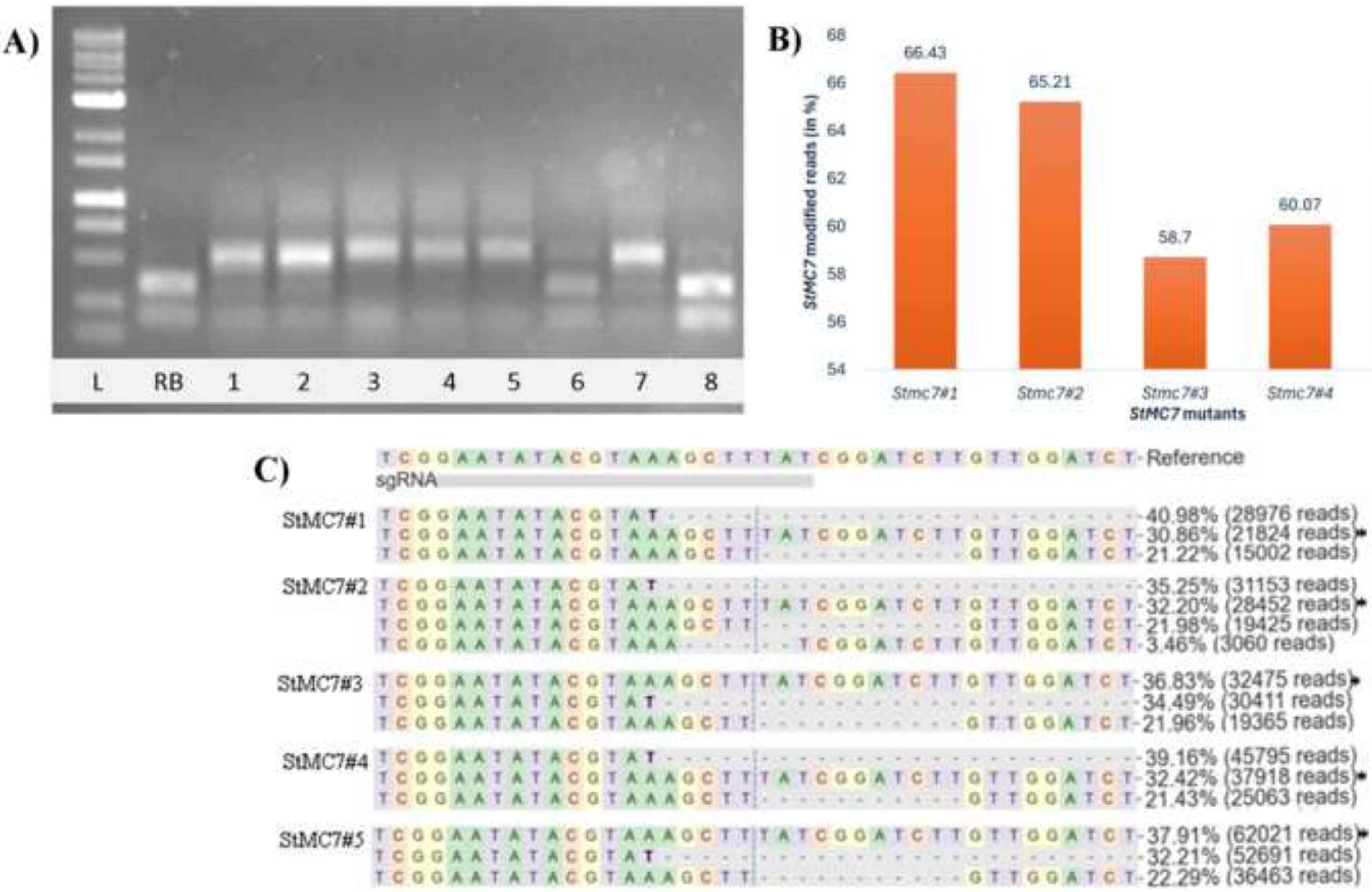

Figure 5 Click here to access/download;Figure;pLOS-one-image-5.tiff

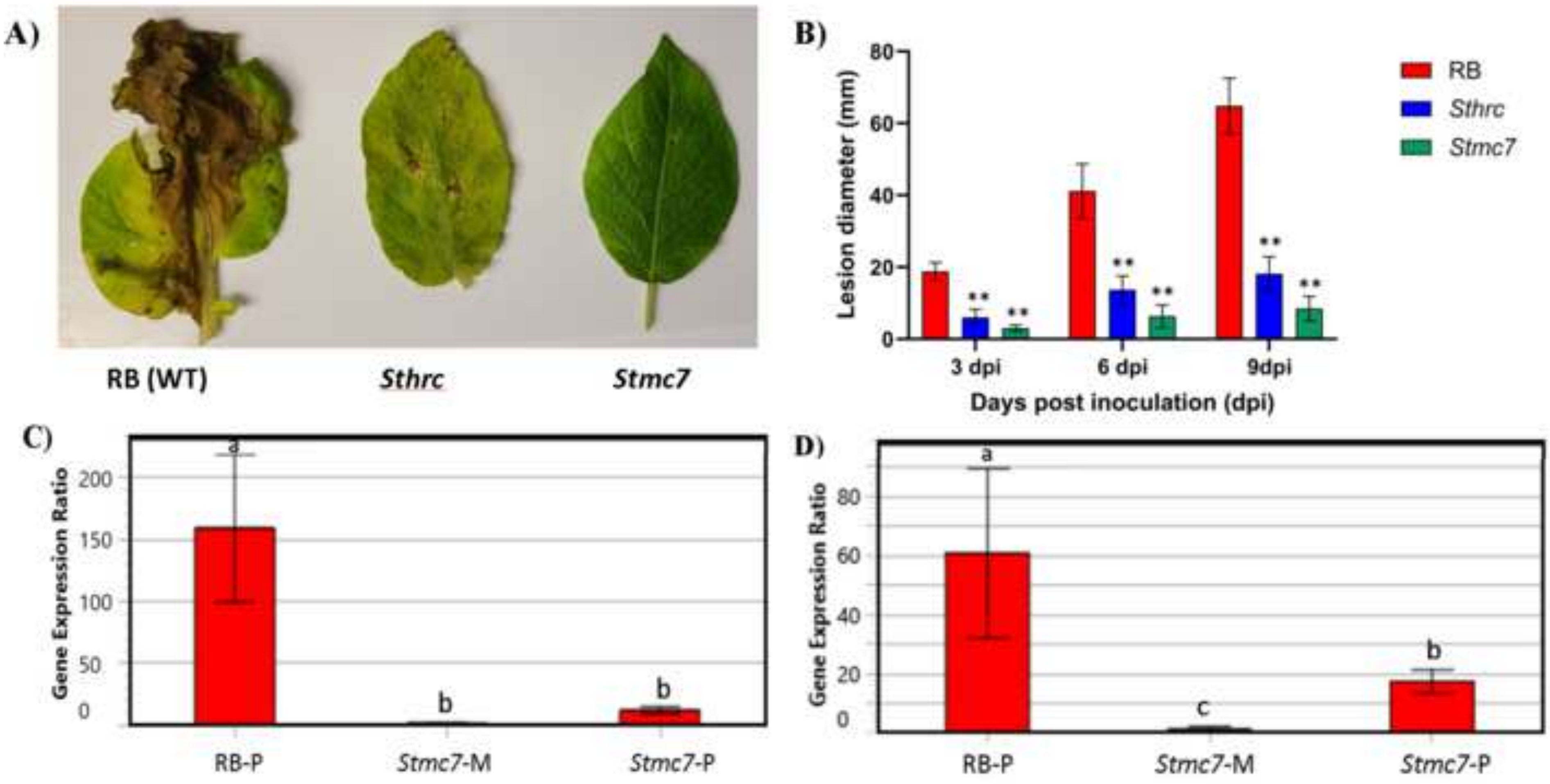

Figure 6 Click here to access/download;Figure;pLOS-one-image-6.tiff

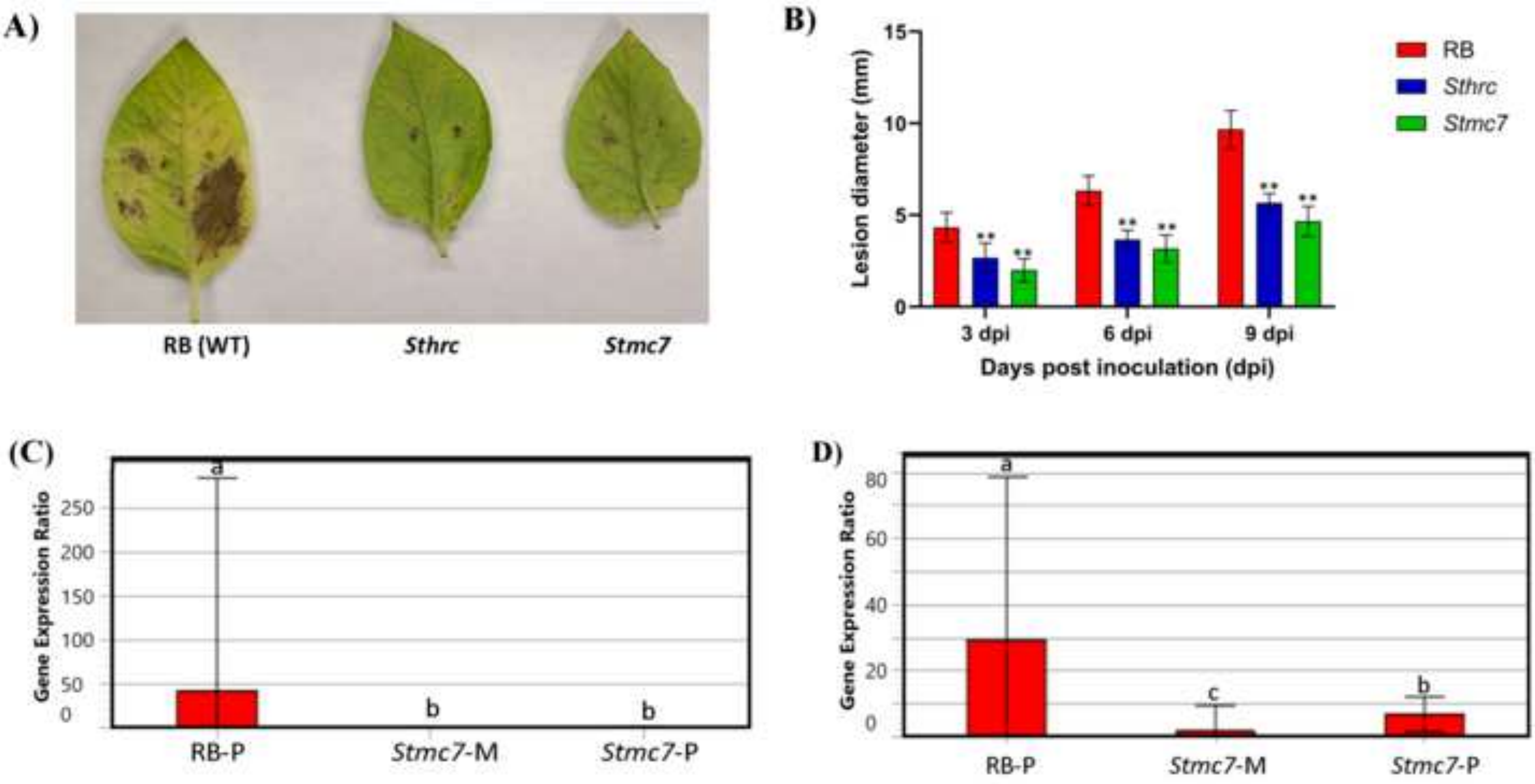

Supplement: Attachment — Submitted filename: PONE-D-25-02422_edited.pdf [file pone.0325702.s001.pdf]
