# Supplementary material for: Editing metacaspase (StMC7) gene enhances late blight resistance in Russet Burbank potato
Source: PLoS One. 2025 Jun 18;20(6):e0325702. doi: 10.1371/journal.pone.0325702 (PMC12176156; doi:10.1371/journal.pone.0325702)
Supplement: Attachment — Submitted filename: Comments_1.pdf [file pone.0325702.s002.pdf]

Dear Editor,

I have examined all the responses provided by the authors and their incorporation into the revised manuscript (ID: PONE-D-25-02422R1), titled "Editing metacaspase (*StMC7*) gene enhances late blight resistance in Russet Burbank potato". The authors have carefully addressed all concerns raised during the initial review. The revised manuscript shows substantial improvement in both clarity and overall quality. I am satisfied with the authors' revisions and responses, and I now recommend the manuscript for publication in its current form.
