# Supplementary material for: Editing metacaspase (StMC7) gene enhances late blight resistance in Russet Burbank potato
Source: PLoS One. 2025 Jun 18;20(6):e0325702. doi: 10.1371/journal.pone.0325702 (PMC12176156; doi:10.1371/journal.pone.0325702)
Supplement: Peer review history

# Author comment: Author response to Decision Letter 0

**DOI:** 10.1371/journal.pone.0325702.r001

17 Jan 2025

# Peer review: Decision Letter 0

**DOI:** 10.1371/journal.pone.0325702.r002

**Authors:** Basavantraya Devanna

24 Feb 2025

PONE-D-25-02422Editing metacaspase (StMC7) gene enhances late blight resistance in Russet Burbank potatoPLOS ONE

Dear Dr. Kushalappa,

Thank you for submitting your manuscript to PLOS ONE. After careful consideration, we feel that it has merit but does not fully meet PLOS ONE’s publication criteria as it currently stands. Therefore, we invite you to submit a revised version of the manuscript that addresses the points raised during the review process.

==============================

Dear Authors, Thank you very much for this interesting article "Editing metacaspase (StMC7) gene enhances late blight resistance in Russet Burbank potato". Before making further decision, I request you to please address the comments from both the reviewers. Regards,

==============================

Please submit your revised manuscript by Apr 10 2025 11:59PM. If you will need more time than this to complete your revisions, please reply to this message or contact the journal office at plosone@plos.org . When you're ready to submit your revision, log on to https://www.editorialmanager.com/pone/ and select the 'Submissions Needing Revision' folder to locate your manuscript file.

Please include the following items when submitting your revised manuscript:

- A rebuttal letter that responds to each point raised by the academic editor and reviewer(s). You should upload this letter as a separate file labeled 'Response to Reviewers'.
- A marked-up copy of your manuscript that highlights changes made to the original version. You should upload this as a separate file labeled 'Revised Manuscript with Track Changes'.
- An unmarked version of your revised paper without tracked changes. You should upload this as a separate file labeled 'Manuscript'.

If you would like to make changes to your financial disclosure, please include your updated statement in your cover letter. Guidelines for resubmitting your figure files are available below the reviewer comments at the end of this letter.

If applicable, we recommend that you deposit your laboratory protocols in protocols.io to enhance the reproducibility of your results. Protocols.io assigns your protocol its own identifier (DOI) so that it can be cited independently in the future. For instructions see: https://journals.plos.org/plosone/s/submission-guidelines#loc-laboratory-protocols . Additionally, PLOS ONE offers an option for publishing peer-reviewed Lab Protocol articles, which describe protocols hosted on protocols.io. Read more information on sharing protocols at https://plos.org/protocols?utm_medium=editorial-email&utm_source=authorletters&utm_campaign=protocols .

We look forward to receiving your revised manuscript.

Kind regards,

Basavantraya N. Devanna, PhD

Academic Editor

PLOS ONE

Journal Requirements:

When submitting your revision, we need you to address these additional requirements.

1. Please ensure that your manuscript meets PLOS ONE's style requirements, including those for file naming. The PLOS ONE style templates can be found at

https://journals.plos.org/plosone/s/file?id=wjVg/PLOSOne_formatting_sample_main_body.pdf and

https://journals.plos.org/plosone/s/file?id=ba62/PLOSOne_formatting_sample_title_authors_affiliations.pdf

2.   Please ensure that the title is match in the manuscript and the cover letter.

3. Thank you for stating the following financial disclosure:

“Weston Loblaws and Natural sciences and engineering council of Canada”

Please state what role the funders took in the study. If the funders had no role, please state: "The funders had no role in study design, data collection and analysis, decision to publish, or preparation of the manuscript."

If this statement is not correct you must amend it as needed.

Please include this amended Role of Funder statement in your cover letter; we will change the online submission form on your behalf.

4. Please include your full ethics statement in the ‘Methods’ section of your manuscript file. In your statement, please include the full name of the IRB or ethics committee who approved or waived your study, as well as whether or not you obtained informed written or verbal consent. If consent was waived for your study, please include this information in your statement as well.

5. Please ensure that you refer to Figure 6 in your text as, if accepted, production will need this reference to link the reader to the figure.

**Additional Editor Comments:**

Dear Authors,

Thank you very much for this interesting article "Editing metacaspase (StMC7) gene enhances late blight resistance in Russet Burbank potato". Before making further decision, I request you to please address the comments from both the reviewers.

Regards,

[Note: HTML markup is below. Please do not edit.]

Reviewers' comments:

Reviewer's Responses to Questions

**Comments to the Author**

1. Is the manuscript technically sound, and do the data support the conclusions?

The manuscript must describe a technically sound piece of scientific research with data that supports the conclusions. Experiments must have been conducted rigorously, with appropriate controls, replication, and sample sizes. The conclusions must be drawn appropriately based on the data presented. 

Reviewer #1: Yes

Reviewer #2: Yes

**********

2. Has the statistical analysis been performed appropriately and rigorously? 

Reviewer #1: Yes

Reviewer #2: Yes

**********

3. Have the authors made all data underlying the findings in their manuscript fully available?

The PLOS Data policy requires authors to make all data underlying the findings described in their manuscript fully available without restriction, with rare exception (please refer to the Data Availability Statement in the manuscript PDF file). The data should be provided as part of the manuscript or its supporting information, or deposited to a public repository. For example, in addition to summary statistics, the data points behind means, medians and variance measures should be available. If there are restrictions on publicly sharing data—e.g. participant privacy or use of data from a third party—those must be specified.

Reviewer #1: Yes

Reviewer #2: Yes

**********

4. Is the manuscript presented in an intelligible fashion and written in standard English?

PLOS ONE does not copyedit accepted manuscripts, so the language in submitted articles must be clear, correct, and unambiguous. Any typographical or grammatical errors should be corrected at revision, so please note any specific errors here.

Reviewer #1: Yes

Reviewer #2: Yes

**********

5. Review Comments to the Author

Please use the space provided to explain your answers to the questions above. You may also include additional comments for the author, including concerns about dual publication, research ethics, or publication ethics. (Please upload your review as an attachment if it exceeds 20,000 characters)

Reviewer #1: Dear Authors,

I have made my comments in the PDF version of the manuscript. The authors are requested to review all comments in the edited version and address them carefully. A graphical representation has been suggested to enhance the understanding of the resistance mechanism of the StMC7 mutant allele in potatoes against pathogens, provide a better reading experience for readers, and improve the quality of the manuscript.

Reviewer #2: Dear Authors,

This is very valuable findings on the editing of StMC7 involved in the PCD pathway which reduces the disease severity of Phytophthora and Alternaria pathogen in potato. The manuscript is well written and easily under stable. The following suggestions may be addressed for improvement in the manuscript. Best wishes

Major comments:

1. The detection of T0 plants positive for transformation with vector specifc/T-DNA specific primers are not mentioned in methodology. PCR screening of T0 lines for HPT or other primers can be included in Figure 3

2. Editing was confirmed using the RD and NGS technology. The wild type amplicon sequencing data was not mentioned in NGS. Further, NGS library details, primers, no. of reads, and read length can be updated in the manuscript. As the authors have detected the editing using restriction enzyme assay, SANGER sequencing could be performed for substantiation.

3. Interesting to note that the expression of the StMC7 was also highly reduced in edited lines. Might be instead of comparison between edited and non transformed wild type, it is suggested to compare between the infected and non-infected leaves of the wild type and edited lines. This reduction could be indirect effect of reduced PCD upon pathogen infection. This needs to be elaborated in the discussion.

Minor Comments

Line No-162: RBD design is done with experimental units separated into blocks, this would be CRD, verify

Line no-199: StMC7, check for italics and throughout the manuscript

Line no 248: P. infestans, check for italics and throughout the manuscript

Figure 6 legends is not complete.

Fig.5 and 6: Gene expression ratio could be changed to relative fold change

Fig.4: Please mention generation of seedlings in which tested, guess it is T0

Liter Units is mentioned as L or l in the manuscript, check for uniformity

Three guides are used and assay of editing is reported for one single guide. The details of editing for other two guide may be included

Conditions for disease screening can be updated as temperature requirement for both the pathogens for causing disease symptoms varies considerably

As resistance is mentioned in the title and the manuscript, disease resistance score (SES 1-9) could be updated in the manuscript (Methodology and Results).

DNA laddering is the characteristics of AL-PCD and it would be interesting to know the status of the DNA laddering in the infected edited leaves

**********

6. PLOS authors have the option to publish the peer review history of their article (what does this mean? ). If published, this will include your full peer review and any attached files.

If you choose “no”, your identity will remain anonymous but your review may still be made public.

**Do you want your identity to be public for this peer review?** For information about this choice, including consent withdrawal, please see our Privacy Policy .

Reviewer #1: **Yes: ** Dr Pankaj Kumar Singh

Reviewer #2: **Yes: ** Parameswaran Chidambaranathan

**********

[NOTE: If reviewer comments were submitted as an attachment file, they will be attached to this email and accessible via the submission site. Please log into your account, locate the manuscript record, and check for the action link "View Attachments". If this link does not appear, there are no attachment files.]

While revising your submission, please upload your figure files to the Preflight Analysis and Conversion Engine (PACE) digital diagnostic tool, https://pacev2.apexcovantage.com/ . PACE helps ensure that figures meet PLOS requirements. To use PACE, you must first register as a user. Registration is free. Then, login and navigate to the UPLOAD tab, where you will find detailed instructions on how to use the tool. If you encounter any issues or have any questions when using PACE, please email PLOS at figures@plos.org . Please note that Supporting Information files do not need this step.

Attachment Submitted filename: PONE-D-25-02422_edited.pdf

# Author comment: Author response to Decision Letter 1

**DOI:** 10.1371/journal.pone.0325702.r003

16 Apr 2025

PLOS One

April 10, 2025

Dear Dr. Devanna and Reviewers,

We are pleased to receive the comments from our reviewers. We believe these feedbacks are very helpful in improving our manuscript and are grateful for the reviews. We have responded to all their comments in detail in the following pages. Reviewers’ comments are in black and our reply to the reviews is in maroon. We would like to thank the reviewers for their critical comments.

Reviewer #1:

Line 30 : Is Stmc7 a knockout mutant or knockdown (or silenced)?

- Agreed. We have modified into “Stmc7 knockdown mutants.”

Line 47: added “as well as”

- Agreed. We have added the phrase "as well as”

Line 69: HRC: What are other functions of this gene rather than involvement in plant resistance against pathogen?

- HRC regulates calcium uptake and release and maintain the calcium ion homeostasis. It may influence the expression of other genes involved in plant defense response. Further study is needed to understand other roles of the gene. The next sentence in line 70 explains the known function of HRC.

Line 92: There are a total of nine metacaspase genes in potato. Why was StMC7 chosen for characterization in this crop?

- We have mentioned the reason for selecting StMC7 in line 85 – 88. In addition, the research article we cited as 20 i.e. Dubey et al. (2019) also demonstrated that StMC7 is the most highly expressed metacaspase gene in most tissues during both biotic and abiotic stress conditions, which made StMC7 a prime candidate gene for silencing. Our previous study demonstrated reduced expression of StMC7 upon silencing StHRC. This was the main reason for selection of this metacaspase.

Line 95: Removed phrase ‘for the pathogen.’

- Corrected.

Line 102: What could be the reason for exploiting this susceptible genotype? Why not use another Indian elite potato cultivar that also shows susceptibility to late blight and early blight pathogens?

- Our research lab is based in North America and Russet Burbank is one of the most desired cultivars among farmers as it is suitable for both fresh and processing markets.

Line 149: Italicize the name of the restriction endonuclease.

- Corrected.

Line 160: Room temperature varies depending on location and geographical conditions. Thus, please provide an approximate incubation temperature for A. solani.

- Agreed. Room temperature of 21 - 23 degree Celsius was added in line 165 for A. solani.

Line 213 (Figure 1 legend) and Line 220 (Figure 4 legend): Italicize the name of the restriction endonuclease.

- Corrected at Line 219, 229.

Line 243 ( Figure 5 legend): Check and correct the formatting style of the paragraph.

- Corrected.

Line 248 (Figure 5 legend): Italicize the scientific name.

- Corrected.

Line 255 (Figure 6 legend): Check and correct the formatting style of the paragraph.

- Corrected.

Line 274: The metacaspase 7 (StMC7) gene was either silenced or knocked out, as mentioned in the heading of the results section, "StMC7 Knockout Based on CRISPR-Cas9". The authors need to confirm whether the StMC7 gene was knocked down, knocked out, or both in the RB potato. Based on this confirmation, they should make the necessary changes across the main text, tables, and figures of the manuscript. To knock down or silence a gene means reducing its expression. To knock out a gene means completely suppressing its expression.

- Agreed. Changed knockout to knockdown or silencing at different parts of manuscript.

o Line 94: Changed ‘knockout’ to ‘silencing’.

o Line 183: Changed ‘knockout’ to ‘knockdown’.

o Line 207: Changed ‘knockout’ to ‘silencing’.

Line 295: Why can't the authors construct a model, incorporating the previous one along with the findings obtained in this study? Generating a graphical representation showing how the StMC7 mutant allele contributes to resistance in potatoes against pathogens would be a better way to enhance readers' understanding and improve the quality of the manuscript.

- Agreed. We have added Figure 7 in the manuscript with the following legend in line 322-334: “Figure 7. Proposed model of apoptotic-like programmed cell death (AL-PCD) in plants. Upon Ca2+ entry associated with pathogen attack, Ca2+ sensors decode the calcium signal for downstream activation. StHRC (histidine-rich calcium-binding protein) binds with Ca2+ and moves into the nucleus, increasing the calcium concentration in nucleus which activates endonucleases and causes DNA fragmentation. Ca2+ also enters the mitochondria through VDAC (voltage-dependent anion channel), which produces ROS in mitochondria. Ca2+ activates metacaspases StMC7 in cytosol. The StMC7 then cleaves PROPEP1, releasing PEP1, which signals nearby cells to initiate damage response and leads to condensed vacuole and other organelles. The major characteristics of AL-PCD are shown in bold letters: Plasma membrane breakdown, condensed organelles such as vacuole, mitochondria, condensed chromosome, membrane blebbing. Abbreviations: CaMs: calmodulins, CDPKs: Calcium-dependent protein kinases, CBLs: calcineurin B-like proteins.”

Line 322: What is the RR metabolite? Please briefly describe it for better clarity.

- Agreed. RR metabolites are resistance related metabolites, biosynthesized following pathogen invasion, and reduce pathogen advancement through host cell wall thickening and their antifungal and/or antioxidant properties. For examples: phenylpropanoids (hydroxycinnamic acid amides, flavonoids, monolignols, aldehydes etc.) Added the following in line 343 – 344 “several resistance related (RR) metabolites important for reducing pathogen advancement through host cell wall accumulation and antifungal properties [48].”

Reviewer #2:

1. The detection of T0 plants positive for transformation with vector specific/T-DNA specific primers are not mentioned in methodology. PCR screening of T0 lines for HPT or other primers can be included in Figure 3

- Agreed. We have added the following in methodology section at line 146 – 149, “Initial screening of transgenic seedlings was conducted through a PCR with Cas9 primers (Cas9-F and Cas9-R) (Table 1) using Phusion Green Hot Start II High-Fidelity PCD Master Mix (ThermoFisher Scientific, MA, USA) and run on 1% agarose gel, revealing a positive 957-bp band for Cas9 positive seedlings.”.

We have added figure 4A, showing the results from PCR screening of Cas9 and added the following to the Figure 4 legend (line 226 - 228) “(A) Confirmation of Cas9 presence in all eight regenerated potato seedlings through a 957 bp band. L: 1 kb plus ladder, +: Positive Control (pDIRECT21A plasmid), 1-8: Stmc7 transgenic samples, RB: wild type”.

- We have also added the following in results (line 213 – 214) “PCR and gel analysis of all regenerated eight seedlings exhibited the presence of Cas9 (Fig 4A).”

2. Editing was confirmed using the RD and NGS technology. The wild type amplicon sequencing data was not mentioned in NGS.

-The mutation analysis conducted based on NGS data for the mutants were compared to the wild type NGS data, shown in Figure 4(C).

Further, NGS library details, primers, no. of reads, and read length can be updated in the manuscript.

- NGS library detail and primers were added in line 153-155 “A barcoding library was prepared using tags NGS-tag-F + StMC7-gRNA-1-F and NGS-tag-R + StMC7-gRNA-1-R. The library was purified and..”. Corresponding to the above sentence, StMC7 amplicon primers in Table 1, which was composed of NGS-tag + StMC7-gRNA-1, was removed and replaced with NGS-tag primers.

As the authors have detected the editing using restriction enzyme assay, SANGER sequencing could be performed for substantiation.

- We initially sent samples for Sanger sequencing. However, we regularly got multiple peaks in edited lines, possibly due to heterozygous mutation, and hence we went for NGS sequencing for a concrete proof of mutation.

3. Interesting to note that the expression of the StMC7 was also highly reduced in edited lines. Might be instead of comparison between edited and non-transformed wild type, it is suggested to compare between the infected and non-infected leaves of the wild type and edited lines. This reduction could be indirect effect of reduced PCD upon pathogen infection. This needs to be elaborated in the discussion.

- We have indeed conducted comparison between infected and non-infected leaves for wild type and edited lines for gene expression study. The relative gene expression for all the groups RB-P (wild type-pathogen inoculated), Stmc7-M (mutant non-infected) and Stmc7-P (mutant-pathogen inoculated) are calculated compared to the wild type non-infected leaves as control.

Minor Comments

Line No-162: RBD design is done with experimental units separated into blocks, this would be CRD, verify.

- The treatments within the blocks were randomized, thus what we have done is RCBD.

Line no-199: StMC7, check for italics and throughout the manuscript

- Corrected.

Line no 248: P. infestans, check for italics and throughout the manuscript

- Corrected.

Figure 6 legends is not complete.

- We intended to exclude our pathogen biomass and gene expression data for Alternaria solani due to high Standard Error of Mean (SEM) in our experiment and hence removed the associated legend but submitted the image with gene expression by mistake. We have uploaded the correct figure, excluding figure C and D.

Fig.5 and 6: Gene expression ratio could be changed to relative fold change

- Agreed. We have changed from gene expression ratio to Relative gene expression. We have also added fold change (FC) value at line 245 - 246 and line 249.

Fig.4: Please mention generation of seedlings in which tested, guess it is T0

- Agreed. Added T0 in figure 4 title.

Liter Units is mentioned as L or l in the manuscript, check for uniformity

- Corrected

Three guides are used and assay of editing is reported for one single guide. The details of editing for other two guide may be included.

- We found editing in only one target and no editing for other two guide RNAs. Hence, we have reported editing in only one gRNA. We have mentioned that we didn’t observe mutations for gRNA 2 and 3 in line 215.

Conditions for disease screening can be updated as temperature requirement for both the pathogens for causing disease symptoms varies considerably.

- Agreed. We have added the following phrase “and kept in greenhouse at 21-23oC.” in line 172. Though the optimum condition for late blight is lower than that of early blight, we were bound by greenhouse temperature condition. The plants were covered in plastic bags to maintain >90% humidity, which is more important for disease progression.

As resistance is mentioned in the title and the manuscript, disease resistance score (SES 1-9) could be updated in the manuscript (Methodology and Results).

- Disease resistance was measured as average lesion diameter, which was used to calculate the area under disease progress curve (AUDPC). We believe this method is more accurate and non-biased. Hence, we believe the measures we used are appropriate to discuss resistance.

DNA laddering is the characteristics of AL-PCD and it would be interesting to know the status of the DNA laddering in the infected edited leaves.

- Agreed. However, we couldn’t conduct DNA laddering experiment because of time constraints. We have conducted DNA laddering experiment in our previous study involving silencing of HRC.

# Peer review: Decision Letter 1

**DOI:** 10.1371/journal.pone.0325702.r004

**Authors:** Basavantraya Devanna

19 May 2025

Editing metacaspase (StMC7) gene enhances late blight resistance in Russet Burbank potato

PONE-D-25-02422R1

Dear Dr. Kushalappa,

We’re pleased to inform you that your manuscript has been judged scientifically suitable for publication and will be formally accepted for publication once it meets all outstanding technical requirements.

Within one week, you’ll receive an e-mail detailing the required amendments. When these have been addressed, you’ll receive a formal acceptance letter and your manuscript will be scheduled for publication.

An invoice will be generated when your article is formally accepted. Please note, if your institution has a publishing partnership with PLOS and your article meets the relevant criteria, all or part of your publication costs will be covered. Please make sure your user information is up-to-date by logging into Editorial Manager at Editorial Manager®  and clicking the ‘Update My Information' link at the top of the page. If you have any questions relating to publication charges, please contact our Author Billing department directly at authorbilling@plos.org.

If your institution or institutions have a press office, please notify them about your upcoming paper to help maximize its impact. If they’ll be preparing press materials, please inform our press team as soon as possible -- no later than 48 hours after receiving the formal acceptance. Your manuscript will remain under strict press embargo until 2 pm Eastern Time on the date of publication. For more information, please contact onepress@plos.org.

Kind regards,

Basavantraya N. Devanna, PhD

Academic Editor

PLOS ONE

Additional Editor Comments (optional):

Reviewers' comments:

Reviewer's Responses to Questions

**Comments to the Author**

1. If the authors have adequately addressed your comments raised in a previous round of review and you feel that this manuscript is now acceptable for publication, you may indicate that here to bypass the “Comments to the Author” section, enter your conflict of interest statement in the “Confidential to Editor” section, and submit your "Accept" recommendation.

Reviewer #1: All comments have been addressed

**********

2. Is the manuscript technically sound, and do the data support the conclusions?

The manuscript must describe a technically sound piece of scientific research with data that supports the conclusions. Experiments must have been conducted rigorously, with appropriate controls, replication, and sample sizes. The conclusions must be drawn appropriately based on the data presented. 

Reviewer #1: Yes

**********

3. Has the statistical analysis been performed appropriately and rigorously? 

Reviewer #1: N/A

**********

4. Have the authors made all data underlying the findings in their manuscript fully available?

The PLOS Data policy requires authors to make all data underlying the findings described in their manuscript fully available without restriction, with rare exception (please refer to the Data Availability Statement in the manuscript PDF file). The data should be provided as part of the manuscript or its supporting information, or deposited to a public repository. For example, in addition to summary statistics, the data points behind means, medians and variance measures should be available. If there are restrictions on publicly sharing data—e.g. participant privacy or use of data from a third party—those must be specified.

Reviewer #1: Yes

**********

5. Is the manuscript presented in an intelligible fashion and written in standard English?

PLOS ONE does not copyedit accepted manuscripts, so the language in submitted articles must be clear, correct, and unambiguous. Any typographical or grammatical errors should be corrected at revision, so please note any specific errors here.

Reviewer #1: Yes

**********

6. Review Comments to the Author

Please use the space provided to explain your answers to the questions above. You may also include additional comments for the author, including concerns about dual publication, research ethics, or publication ethics. (Please upload your review as an attachment if it exceeds 20,000 characters)

Reviewer #1: I have examined all the responses provided by the authors and their incorporation into the revised manuscript (ID: PONE-D-25-02422R1), titled "Editing metacaspase (StMC7) gene enhances late blight resistance in Russet Burbank potato". The authors have carefully addressed all concerns raised during the initial review. The revised manuscript shows substantial improvement in both clarity and overall quality. I am satisfied with the authors’ revisions and responses.

**********

7. PLOS authors have the option to publish the peer review history of their article (what does this mean? ). If published, this will include your full peer review and any attached files.

If you choose “no”, your identity will remain anonymous but your review may still be made public.

**Do you want your identity to be public for this peer review?** For information about this choice, including consent withdrawal, please see our Privacy Policy .

Reviewer #1: **Yes: ** Dr Pankaj Kumar Singh

**********

Attachment Submitted filename: Comments_1.pdf

# Editor report: Acceptance letter

**DOI:** 10.1371/journal.pone.0325702.r005

**Authors:** Basavantraya Devanna

PONE-D-25-02422R1

PLOS ONE

Dear Dr. Kushalappa,

I'm pleased to inform you that your manuscript has been deemed suitable for publication in PLOS ONE. Congratulations! Your manuscript is now being handed over to our production team.

At this stage, our production department will prepare your paper for publication. This includes ensuring the following:

* All references, tables, and figures are properly cited

* All relevant supporting information is included in the manuscript submission,

* There are no issues that prevent the paper from being properly typeset

You will receive further instructions from the production team, including instructions on how to review your proof when it is ready. Please keep in mind that we are working through a large volume of accepted articles, so please give us a few days to review your paper and let you know the next and final steps.

Lastly, if your institution or institutions have a press office, please let them know about your upcoming paper now to help maximize its impact. If they'll be preparing press materials, please inform our press team within the next 48 hours. Your manuscript will remain under strict press embargo until 2 pm Eastern Time on the date of publication. For more information, please contact onepress@plos.org.

If we can help with anything else, please email us at customercare@plos.org.

Thank you for submitting your work to PLOS ONE and supporting open access.

Kind regards,

PLOS ONE Editorial Office Staff

on behalf of

Dr. Basavantraya N. Devanna

Academic Editor

PLOS ONE
